# High-Mobility Hole Transport in Single-Grain PbSe Quantum Dot Superlattice Transistors

**DOI:** 10.1021/acs.nanolett.2c03657

**Published:** 2022-11-21

**Authors:** Alex Abelson, Caroline Qian, Zachary Crawford, Gergely T. Zimanyi, Matt Law

**Affiliations:** † Department of Materials Science and Engineering, University of California, Irvine, Irvine, California 92697, United States; ‡ Department of Chemical and Biomolecular Engineering, University of California, Irvine, Irvine, California 92697, United States; § Department of Chemistry, University of California, Irvine, Irvine, California 92697, United States; ∥ Department of Physics, University of California, Davis, Davis, California 95616, United States

**Keywords:** colloidal quantum dots, superlattice, PbSe, single grain, field-effect transistor, charge transport

## Abstract

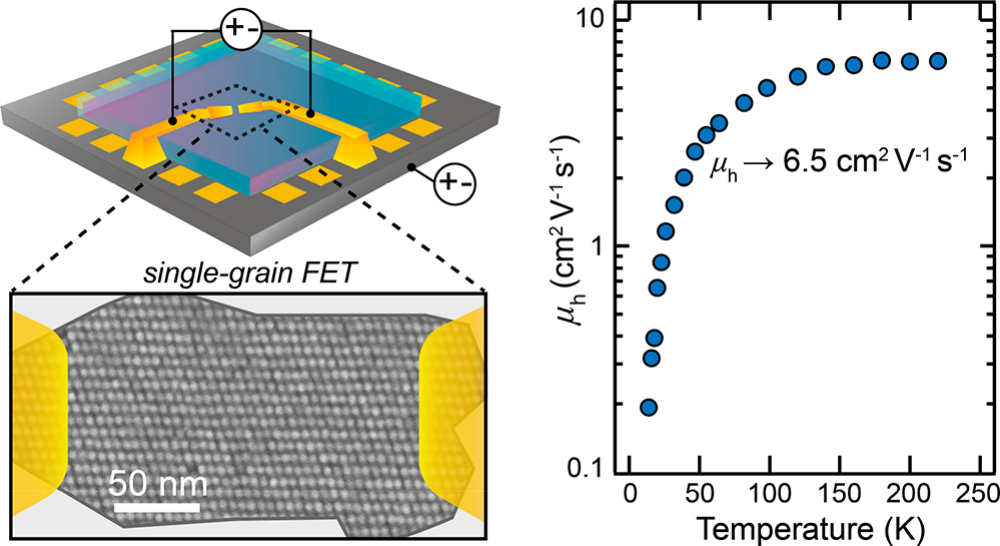

Epitaxially-fused superlattices of colloidal quantum dots (QD epi-SLs) may exhibit electronic minibands and high-mobility charge transport, but electrical measurements of epi-SLs have been limited to large-area, polycrystalline samples in which superlattice grain boundaries and intragrain defects suppress/obscure miniband effects. Systematic measurements of charge transport in individual, highly-ordered epi-SL grains would facilitate the study of minibands in QD films. Here, we demonstrate the air-free fabrication of microscale field-effect transistors (μ-FETs) with channels consisting of single PbSe QD epi-SL grains (2–7 μm channel dimensions) and analyze charge transport in these single-grain devices. The eight devices studied show *p*-channel or ambipolar transport with a hole mobility as high as 3.5 cm^2^ V^–1^ s^–1^ at 290 K and 6.5 cm^2^ V^–1^ s^–1^ at 170–220 K, one order of magnitude larger than that of previous QD solids. The mobility peaks at 150–220 K, but device hysteresis at higher temperatures makes the true mobility–temperature curve uncertain and evidence for miniband transport inconclusive.

Epitaxially-fused colloidal PbX (X = S, Se) quantum dot (QD) superlattices (epi-SLs)—arrays of QDs interconnected by homoepitaxial necks—combine extraordinary spatial order with strong inter-QD electronic coupling and are predicted to exhibit electronic minibands that enable high-mobility, bandlike charge transport.^1−12^ However, the epi-SLs studied thus far are too structurally defective (disordered) to support miniband formation, and electrical measurements of epi-SLs have demonstrated only localized carriers and hopping transport.^7,8^ Ongoing efforts to eliminate intragrain defects are essential to trigger the emergence of minibands,^12−14^ but it is also important to remove intergrain defects (grain boundaries and cracks) from the transport path in order to study the *inherent* properties of individual epi-SL grains (monocrystals).^15^ A robust process for making measurements at the single-grain limit (monocrystalline devices) would enable systematic studies of miniband physics as a function of epi-SL structural order, surface chemistry, orientation, device size, and other parameters.

In this report, we describe the fabrication and electrical characterization of single-grain PbSe QD epi-SL field-effect transistors (FETs) made by multistep electron beam lithography (EBL). We infill and overcoat the epi-SLs with a thin layer of amorphous alumina by atomic layer deposition (ALD)^16,11^ to prevent their degradation by exposure to air, processing chemicals, mild heating, and the electron beam. We find that the mild heating needed to bake the electron-beam resist for EBL processing (150 °C) also drastically lowers the doping of the ALD-infilled epi-SLs, without causing QD sintering. Variable-temperature electrical measurements of the resulting single-grain FETs (12–290 K) show a record-high hole mobility with a negative temperature dependence at higher temperatures that is suggestive of bandlike transport, but this antiactivated region is almost certainly an artifact of the bias-stress effect^17^ rather than a signature of minibands. This paper introduces the single-grain FET fabrication process, details the structure and electrical properties of one device, briefly compares the behavior of an initial cohort of eight devices, and concludes with an analysis of the temperature dependence of the hole mobility in the four devices that were studied in detail.

3D (multilayer) epi-SL films were fabricated by self-assembly of 6.9 nm diameter oleate-capped PbSe QDs (Figure S1) on the surface of liquid ethylene glycol (EG),^18^ followed by injection of 1,2-ethylenediamine to trigger epitaxial fusion (necking) of the QDs via glycoxide–oleate ligand exchange^11,12^ and stamp transfer of the resulting polycrystalline epi-SLs to custom-made device substrates (see Methods in the Supporting Information and Figure 1a). This procedure yields epi-SLs with a rhombohedrally-distorted simple cubic unit cell (*a* ≈ 6.9 nm, α ≈ 99°) in which the QDs are necked across their {100} facets and the epi-SL grains are oriented predominantly with their (011̅)_SL_ planes parallel to the substrate surface (Figure 1b).^11^ Scanning electron microscopy (SEM) imaging showed that each epi-SL film consists of a cracked, semicontinuous layer of equiaxed epi-SL grains (individual epi-SL crystallites) with a grain diameter as large as 10 μm (Figure 2a). Film thicknesses of ∼35 nm (“thin”) and 60–85 nm (“thick”) were studied. As discussed below, the thinner films show significantly higher hole mobility, probably because they are more fused and have thicker inter-QD necks.

**Figure 1:**
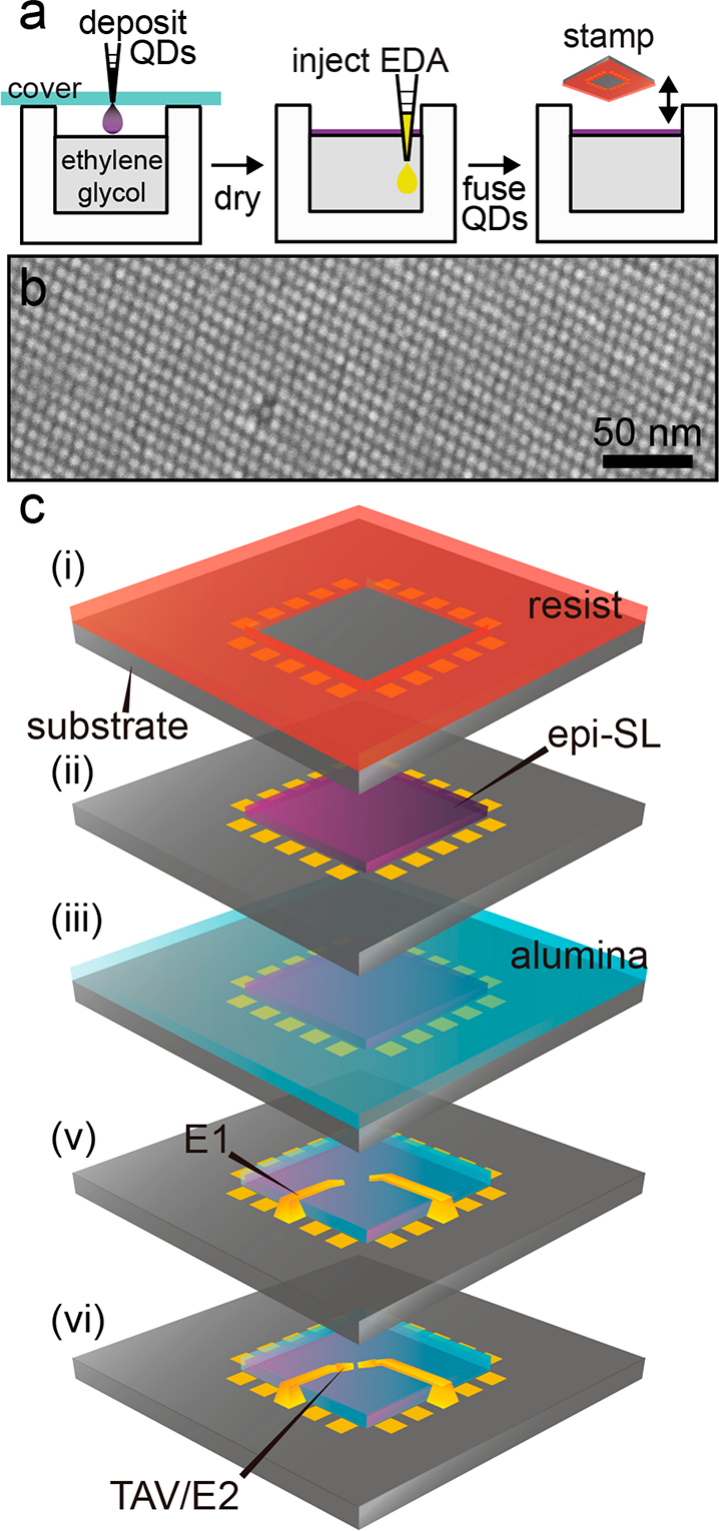
Fabrication of single-grain epi-SL field-effect transistors. (a) Process for fabricating polycrystalline epi-SL films on the surface of ethylene glycol. (b) Post-mortem high-magnification SEM image of part of an epi-SL grain. The scale bar is 50 nm. (c) The single-grain FET fabrication process. (i) The Si/SiO_2_ substrate (gray) was first patterned with photoresist (red) to expose a 550 × 550 μm square at the center of an array of prepatterned contact pads (gold). (ii) A polycrystalline epi-SL film was stamped onto the substrate and the resist was dissolved, leaving epi-SL film (purple) only in the central square. (iii) Alumina (blue) was deposited by ALD on the entire substrate, thereby infilling and overcoating the epi-SL. The substrate was then imaged by SEM to locate epi-SL grains of interest. (v) Coarse electrodes (E1) were written by EBL from the contact pads to within several micrometers of each selected epi-SL grain. (vi) Finally, through-alumina vias (TAVs) were patterned by EBL, wet-etched to expose the edges of each epi-SL grain and E1 electrode, and metalized (E2) to complete the single-grain epi-SL FETs. The image in (b) was acquired after FET fabrication and a post-mortem etch of the alumina to reveal the QDs.

**Figure 2:**
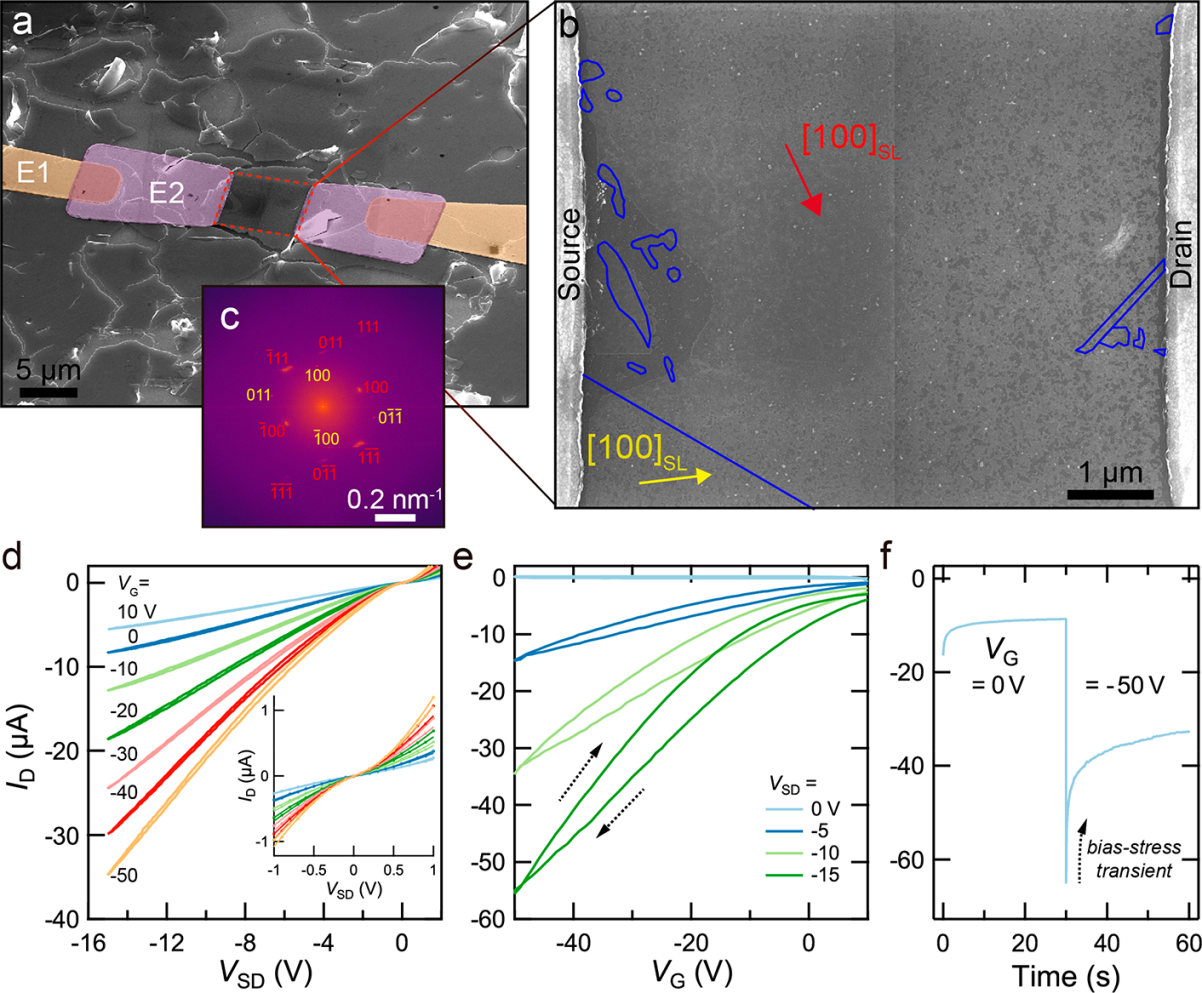
A single-grain epi-SL FET. (a) Perspective-view SEM image of a completed single-grain FET (Device 1, with channel dimensions of 6.8 × 5.9 μm). E1 and E2 electrodes are colorized and labeled. (b) High-resolution plan-view SEM image of the FET channel after a post-mortem etch of the alumina coating. E2 source and drain electrodes are labeled. Blue lines denote SL twin planes. The [100]_SL_ SL direction of the major epi-SL grain (red) and minor grain (yellow) are labeled. There are no additional planar or linear SL defects visible in the image. The faint vertical line near the center of the image is an image stitching artifact. (c) FFT of the entire channel with indexed SL spots for the major (red) and minor (yellow) epi-SL grains. (d) Room-temperature output characteristics (*I*_D_–*V*_SD_) showing modulation of the drain current (*I*_D_) by the applied gate bias (*V*_G_) and *p*-channel behavior. The inset shows that the curves are nonlinear near *V*_SD_ = 0 V. At each *V*_G_ (starting with *V*_G_ = 10 V), *V*_SD_ was swept at a rate of 5 V/s from +10 to −50 V, then back to 10 V. (e) Room-temperature transfer characteristics (*I*_D_–*V*_G_) for forward and reverse gate bias sweeps (sweep directions noted by black arrows). At each *V*_SD_ (starting with *V*_SD_ = 10 V), *V*_G_ was swept at a rate of 200 V/s from +10 to −50 V, then back to 10 V. (f) Room-temperature *I*_D_ time trace upon stepping *V*_G_ from 0 to −50 V at *t* = 30 s (*V*_SD_ = −15 V). The FET exhibits a significant bias-stress-effect transient at room temperature.

Figure 1c depicts the air-free single-grain FET fabrication process that we developed to deterministically incorporate selected high-quality epi-SL grains into FETs to study charge transport at the single-grain limit. First, PbSe QD epi-SL films were stamped onto prepatterned substrates (*p*^++^ (100)-oriented Si coated with a 200 nm thick SiO_2_ layer and patterned with an array of metal contact pads and fiducial markers (5 nm Cr/45 nm Au)) and then infilled and overcoated with 11 nm of amorphous alumina using an in-glovebox atomic layer deposition (ALD) system (see Methods) to prevent epi-SL oxidation/degradation.^16^ Next, the films were imaged by SEM to identify individual high-quality epi-SL grains for selective integration into FETs. The alumina layer thickness was optimized to balance film stability (as validated by FET measurements) and transparency to the electron beam (Figure S2). After imaging, an additional 22 nm of alumina was deposited to ensure indefinite environmental stability of the epi-SLs. We then used EBL to write “coarse” Cr/Au electrodes (labeled E1 in Figure 1c) from the prepatterned contact pads to within a few micrometers of each epi-SL grain of interest. These electrodes sit atop the 33 nm protective alumina layer and are therefore electrically isolated from the underlying film and produce negligible interelectrode leakage currents. Next, EBL-defined through-alumina vias (TAVs) were etched at a rate of ∼0.6 nm/min using an aqueous sodium hydroxide solution (Figure S3) to expose the adjacent edges of the E1 electrodes and targeted grains. The TAVs were then metalized with “fine” Cr/Au electrodes (labeled E2) to connect the E1 electrodes selectively to each respective grain and define the FET channels. Finally, an additional 11 nm or more of alumina was deposited on the finished FETs to ensure their stability. This fabrication process prevents exposure of the QDs to air and lithographic processing chemicals and yields FETs with well-defined channels across single epi-SL grains in excellent electrical isolation from the rest of the film. A detailed description of the single-grain FET fabrication process is provided in Supporting Discussion 1.

Figure 2a shows a 35 nm thick single-grain FET with a channel length (*L*) and width (*W*) of 6.8 and 5.9 μm, respectively. High-resolution SEM imaging (Figure 2b and Figure S4) shows that the channel contains two large (011̅)_SL_-oriented epi-SL grains: a “major” grain that spans the electrodes, covers ∼92% of the channel area, and contains 14 small, isolated twinned inclusions (blue lines in Figure 2b) and a “minor” grain that is adjacent to part of the source and does not span the electrodes. Based on this grain map, we expect that the source-drain current of this device will flow predominantly through the single-crystalline path provided by the major grain, and we therefore consider the device (Device 1) to be a single-grain FET. We used fast Fourier transform (FFT) images of the channel to determine epi-SL grain orientations and inter-QD distances. The FFT image of the entire channel yields a spot pattern that was indexed to the major (red) and minor (yellow) epi-SL grains (Figure 2c), while numerous FFTs of regions within the channel show an average inter-QD distance of 6.86 nm along [100]_SL_ and a planar QD density of 17,390 QDs/μm^2^ per QD monolayer, consistent with our previous reports.^11,12^ Structural analysis of seven additional devices is provided in the Supporting Information, along with the corresponding charge transport data (Figures S5–S18). Devices 1–7 are single-grain FETs, while Device 8 is multicrystalline and serves as a control for the effect of grain boundaries on charge transport. Table S1 summarizes the microstructure and transport data for all eight devices. The seven single-grain FETs studied here are free of the SL grain boundaries and macroscopic cracks/voids that are common in polycrystalline epi-SL films made by ligand exchange and stamp transfer.

At room temperature, all but one of the FETs (Device 6, which is ambipolar) show *p*-channel conductance with moderate gate modulation of the drain current (*I*_D_) and quasi-linear output curves (*I*_D_–*V*_SD_) between 0 and −15 V (Figure 2d). Within *V*_SD_ = ±1 V, the *I*–*V* curves are nonlinear (Figure 2c, inset), likely because of Schottky barriers at the QD/electrode interfaces. The nonlinearity also suggests that the contact resistance in these devices is significant and artificially lowers the values of the carrier mobility that are reported below. Contact resistance measurements and the associated correction of the mobility values will be performed with future batches of single-grain FETs. Transfer curves (*I*_D_–*V*_G_) exhibit hysteresis between forward and reverse *V*_G_ sweeps. Reverse and forward sweeps for Device 1 yield a hole mobility of 4.3 and 6.8 cm^2^ V^–1^ s^–1^ at *V*_G_ = −45 V and *V*_SD_ = −15 V (Figure 2e; see Methods). Hysteresis in the transfer curves is caused by the bias-stress effect (BSE), in which a buildup of a sheet of immobile charges near the gate/channel interface progressively screens the applied gate field and causes a time-dependent (transient) reduction of the free carrier density and *I*_D_ in the FET channel.^17^Figure S19 provides graphical illustrations of the BSE and its electronic and ionic mechanisms. One consequence of the BSE is systematic underestimation of the carrier mobility derived from *I*–*V* curves. To approximately correct the mobility for the BSE, we measured the kinetics of *I*_D_ decay after stepping *V*_G_ from 0 to −50 V at the measurement temperature (Figure 2f and Figure S20) and used the fractional *I*_D_ decay at the mobility measurement time to determine a multiplicative correction factor, yielding a “transient-corrected” reverse and forward sweep mobility of 5.6 and 9.1 cm^2^ V^–1^ s^–1^, respectively, for this device. We note that the true transient-free mobility is probably modestly higher than these imperfectly-corrected values. It is also important to point out that Device 1 consists of an isolated epi-SL flake that barely extends outside of the channel (Figure 2a); thus, fringing electric fields (spreading currents), which can cause mobility overestimation by up to a factor of ∼2 for FETs with *W*/*L* ≈ 1 and semiconductor layers that are much wider than the channel,^19^ are not important and do not affect the mobility for this device. Some of the other seven devices have geometries that may support substantial fringing currents (Figure S21), but we do not attempt to correct the extracted mobility values for this geometric effect because (i) the error in the mobility is small and uncertain, and (ii) the contact resistance and incomplete transient correction cause an uncertain and possibly larger error in the opposite direction.

These results are in contrast to our recent report of degenerate *n*-channel transport in polycrystalline, ALD-infilled epi-SL FETs.^11^ In that work, we found that infilling epi-SLs with ALD alumina generates a large concentration of shallow donor defects that cause heavy electron doping of the films. We have since discovered that these donors can be passivated (or perhaps compensated) by mild thermal annealing of the infilled epi-SLs under either an inert atmosphere or air (Figure S22). Conveniently, the heating steps used to prepare the EBL resist layers (one 30 min dwell at 90 °C and two 3 min dwells at 150 °C) during FET fabrication also reproducibly passivate/compensate the shallow donors and convert the FETs from poorly modulated *n*-channel devices to well-modulated *p*-channel devices. These data suggest that the *p*-channel behavior of these PbSe QD FETs results from annealing rather than air exposure, the latter of which is well-known to *p*-dope PbSe QD films.^16^ However, since the annealing was performed in air, we cannot rule out the possibility of some air-induced oxidation of the heated films. An analysis of post-mortem SEM images (Figures 1b and 2b and Figures S23 and S24) and optical spectra (Figure S22) shows that the annealing does not cause appreciable QD sintering/coarsening or loss of quantum confinement. We therefore believe that the observed changes in FET free carrier density and polarity are primarily caused by annealing-induced chemical reactions, atomic motion, and/or ligand rearrangements at the QD/alumina, QD/electrode and QD/SiO_2_ interfaces. The annealing may also form/thicken epitaxial necks and improve their atomic coherence and thus the inter-QD electronic coupling by healing dislocations and relaxing strain defects,^13,14^ despite encapsulation of the QDs by the conformal alumina coating.

Variable-temperature electrical measurements were used to study charge transport in the single-grain FETs. Figure 3 shows data for Device 1, while results from the other seven devices are provided in the Supporting Information. Decreasing the measurement temperature leads to a shift from *p*-channel to ambipolar transport and a sharp increase in the on–off ratio (from ∼35 at 290 K to >10^6^ at 12 K), consistent with the freeze-out of acceptor states and the influence of Schottky barriers at the contacts (Figure 3a). The output curves (Figure 3b–e) display decreasing conductivity as the device is cooled. Transfer curves (Figure 3f–i) show that the threshold voltage monotonically shifts from positive values at room temperature to negative values (*V*_T_ ≈ −10 V) at 12 K. Furthermore, since the electron/hole trapping and ion motion that cause bias-stress transients are generally thermally activated processes, the BSE is often progressively quenched at low temperatures. Below 150–200 K, the BSE current transients (Figure 3j,k) and *I*–*V* hysteresis are largely quenched and have only a minor impact on the transfer curves and measured carrier mobility.

**Figure 3:**
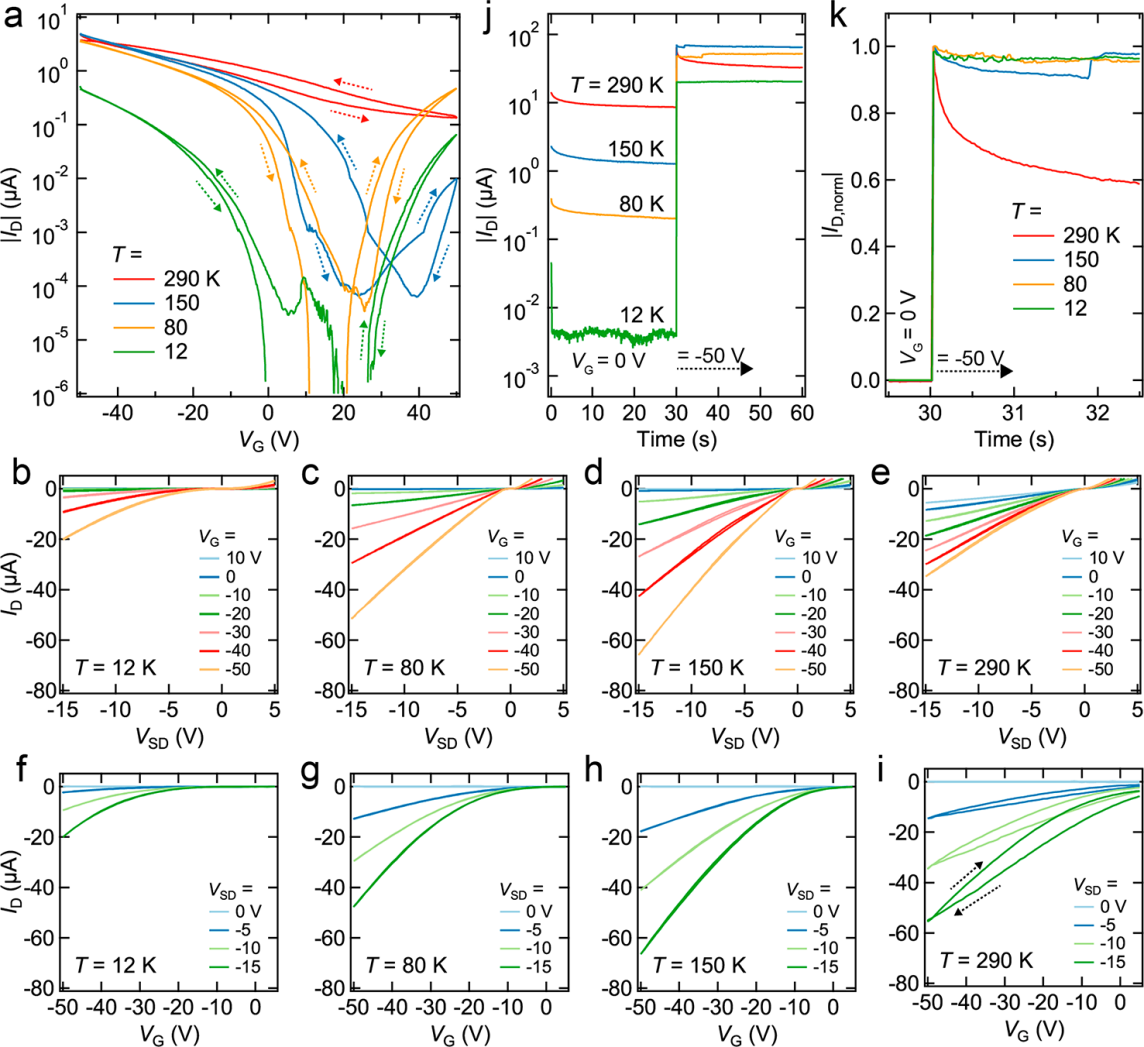
Temperature dependence of the FET *I*–*V* characteristics. (a) Full-sweep (*V*_G_ = ± 50 V) transfer curves for Device 1 acquired at *T* = 12, 80, 150, and 290 K at *V*_SD_ = 2 V and a sweep rate of 5 V/s. The device is a *p*-channel at room temperature and becomes increasingly ambipolar at lower temperature. *I*_OFF_ drops dramatically with decreasing temperature. (b–e) Output curves acquired at *T* = 12, 80, 150, and 290 K, respectively, at a series of *V*_G_ values and a *V*_SD_ sweep rate of 5 V/s. *V*_SD_ was swept from +5 V to −15 V and then back to +5 V. (f–i) Corresponding transfer curves acquired at a series of *V*_SD_ values using a sweep rate of 200 V/s. *V*_G_ was swept from +10 V to −50 V, then back to +10 V. (j) Time traces of |*I*_D_| as *V*_G_ is stepped from 0 to −50 V. Here, *V*_SD_ = −15 V. (k) Comparison of the normalized |*I*_D_| time traces from (j), with , where *I*(*t*) is the drain current at time *t*, *I*_*V*_G_=0_ is the drain current at *V*_G_ = 0 V, and *I*_peak_ is the peak drain current.

Hole mobility (μ_h_) values were extracted from transfer curves acquired at *T* = 12–290 K (see Methods). Figure 4a shows the forward and reverse sweep mobilities for Device 1 with and without correction for bias-stress transients. Below ∼150 K, the transient is negligible and the mobility values converge to a common weakly thermally-activated (dμ/d*T* > 0) curve. The uncorrected data (squares in Figure 4a) show a peak mobility at ∼150 K and a negative temperature dependence (dμ/d*T* < 0) at higher temperatures that is suggestive of bandlike transport,^20−23^ but this is likely an artifact of the bias-stress transients. The corrected data (circles) still show a dμ/d*T* < 0 region, but we cannot be certain if such behavior is real or the result of incomplete transient correction. Given this uncertainty and the small change of mobility with temperature (<2.5×), no conclusion can be made from the transport data regarding the presence of bandlike transport in this FET. All of the devices that we analyzed showed similar μ(*T*) behavior (Figures S25 and S26). The typical μ(*T*) behavior of bulk PbSe is shown in Figure S27 for comparison.

**Figure 4:**
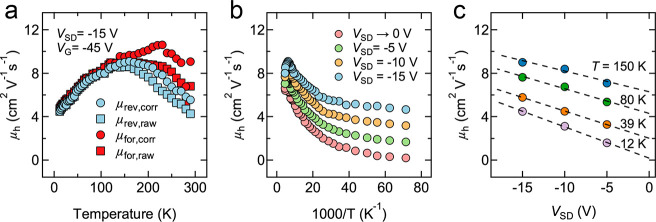
Temperature and field dependence of the hole mobility. (a) Hole mobility (*V*_SD_ = −15 V, *V*_G_ = −45 V) of Device 1 extracted from forward and reverse gate bias sweeps (200 V/s) without transient correction (squares) and with transient correction (circles). The mobility values converge below ∼150 K because the bias-stress transient is quenched. (b) μ_rev,corr_ determined at different *V*_SD_ values plotted as a function of inverse temperature to highlight the dependence of the low-temperature mobility on the longitudinal electric field. The field-free mobility curve (*V*_SD_ → 0 V, pink markers) was determined from the linear fits of μ(*V*_SD_) presented in (c). *V*_G_ = −45 V. (c) Linear fits of μ_rev,corr_ as a function of *V*_SD_. While only four temperatures are shown in (c), fits were performed across the entire temperature range (12–290 K) to produce the field-free mobility curve in (b). *V*_G_ = −45 V.

The hole mobility also depends on *V*_SD_, with the dependence being strongest at low temperature (Figure 4b). Plots of mobility versus *V*_SD_ reveal a linear μ(*V*_SD_) dependence across the entire temperature range (Figure 4c and Figure S26). Linear fits of μ(*V*_SD_) were used to extrapolate a “field-free” mobility (*V*_SD_ → 0 V), which is also plotted in Figure 4b. In short-channel FETs, the longitudinal electric field strength can result in a potential drop between neighboring QD centers (*V*_QD-QD_) equal to or larger than the thermal energy *kT*, the charging energy *E*_C_, and the site energy disorder Δα due to QD size dispersity and coupling disorder. In this case, the mobility can depend strongly on the magnitude of *V*_QD-QD_ rather than *kT*, *E*_C_, and Δα.^24^ For our epi-SLs, we calculate *E*_C_ ≈ 0.3 meV and Δα > 10 meV (see Supporting Discussion 2).^25^*V*_QD-QD_ can be estimated from *V*_SD_ and the number of QDs along the transport path. If transport occurs predominantly in the QD monolayer closest to the gate dielectric, as is expected for FETs with conventional mexal-oxide gates, then the transport path is determined by the length of the epi-fused QD chains that run in the [100]_SL_ direction of the (011̅)_SL_-oriented grains (Figure 1b). SEM imaging shows that the epi-SL grain in Device 1 has a misorientation angle ϕ of 64° between its [100]_SL_ and the FET channel (see Figure 2b and Table S1), such that carriers would need to travel through chains of ∼2250 QDs to transit the channel. Assuming negligible potential drop at the contacts (an idealization), this yields *V*_QD-QD_ values of 2.2, 4.4, and 6.6 mV for *V*_SD_ = −5, −10, and −15 V, respectively. In devices where the epi-fused QD chains are better aligned with the channel, the channel length is shorter, or the second QD monolayer participates in transport (making transport isotropic in 2D), *V*_QD-QD_ can be 20–30 meV at *V*_SD_ = −15 V. Given that *V*_QD-QD_ is similar to or larger than *kT*, *E*_C_, and Δα, a field-dependent mobility is expected, and the same μ(*V*_SD_) trend was observed in the three other devices that we analyzed (Figures S25 and S26). Henceforth we focus only on the field- and transient-corrected mobility values.

The field-free, transient-corrected, reverse-swept hole mobility of our best device (Device 1) is 3.5 cm^2^ V^–1^ s^–1^ at room temperature. This value is a conservative lower bound that does not fully correct for the bias-stress transient or account for possible potential drops at the source/drain contacts. The hole mobility peaks at ∼6.5 cm^2^ V^–1^ s^–1^ at *T* = 170–220 K, where the transient (hysteresis) is insignificant. To our knowledge, these are the highest hole mobilities yet reported for any QD solid, including PbX (previous record of μ_h_ ≈ 0.5 cm^2^ V^–1^ s^–1^),^8,26−30^ CdX, HgX,^23^ CuInSe_*x*_S_2–*x*_,^31^ and metal halide perovskite QDs,^32^ which illustrates the importance of employing epitaxial connections for strong inter-QD coupling, controlled surface chemistry for doping and surface state management, and single-grain measurements to avoid transport bottlenecks by grain boundaries.

We analyzed the μ(*T*) data of Devices 1–3 and 5 (the four devices analyzed in detail) with models of nearest-neighbor hopping (NNH), Efros–Shklovskii variable-range hopping (ES-VRH),^33^ quantum percolation scaling (QPS),^34^ and mixed conduction transport (a limit of QPS).^23^ Due to the presence of current transients at higher temperatures, we limited our analysis to *T* ≤ 220 K, which excludes the dμ/d*T* < 0 region. The simple Arrhenius NNH expression μ(*T*) = μ_∞_ exp(−*E*_A_/*kT*) with a temperature-independent prefactor μ_∞_ yields good fits with small activation energies of only 4–8 meV (Figure 5a and Table 1). These small activation energies may indicate that carriers find low-barrier percolative pathways through the epi-SLs.^25^ Good fits were also obtained with the ES-VRH formula μ(*T*) ∼ (1/*T*) exp(−(*T*_ES_/*T*)^1/2^),^33^ which yielded localization lengths ξ = 6.1*e*^2^/(4πε_eff_ε_0_*k*_B_*T*_ES_) of just 2–4 nm, suggesting that hole wave functions are localized to single QDs and ES-VRH does not occur in these samples (Figure S28, Table 1, and Supporting Discussion 3). Including the normal *T*^–1^ dependence of the prefactor in the NNH expression^35^ gave poor fits that severely underestimated the mobility at low temperature (Figure S29).

**Figure 5:**
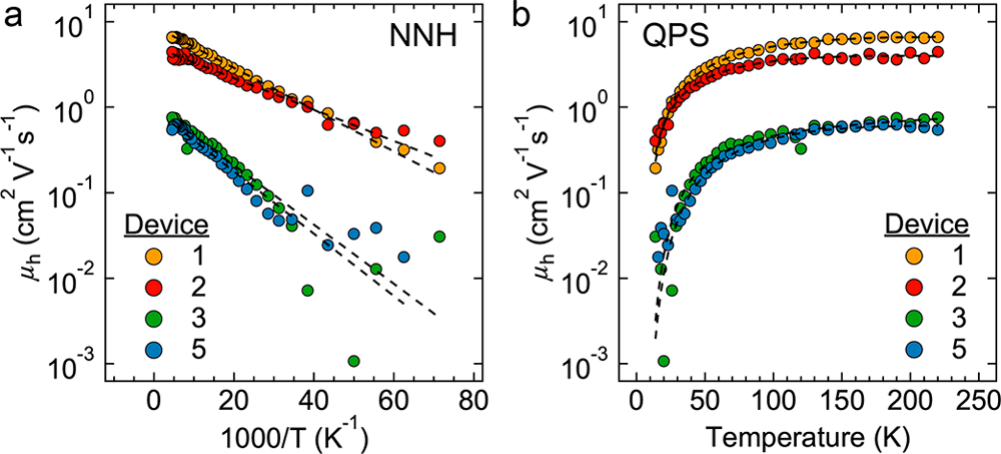
μ_h_(*T*) fitting of four single-grain FETs. (a) NNH model fits. (b) QPS model fits. Fits were performed between 14 and 220 K. Data at 12 K were not included because the field-free mobility values at this temperature tended to be noisy, and >220 K data were excluded because of the BSE.

**Table 1:** Results of μ(*T*) Fitting

	NNH		VRH	QPS	mixed conduction
device	*E*_A_ (meV)	μ_**∞**_ (cm^2^ V^–1^ s^–1^)	ξ (nm)/*T*_ES_ (K)	*E*_A_ (meV)/Δ	ξ (nm)/*T*_ES_ (K)
1	4.6 ± 0.1	8.6 ± 0.1	2.8 / 1000	2.5 / 0.05	10 / 281
2	3.9 ± 0.2	5.2 ± 0.3	3.6 / 792	2.0 / 0.06	15 / 189
3	7.5 ± 0.2	1.1 ± 0.1	2.1 / 1356	4.8 / 0.10	7.6 / 371
5	7.1 ± 0.4	1.1 ± 0.1	2.0 / 1395	4.2 / 0.22	4.1 / 686

Quantitatively better fits were obtained using the quantum percolation scaling (QPS) model of Qu et al.^34^ In this model, carriers delocalize within metallic clusters of well-coupled QDs characterized by a mobility μ_met_^–1^ = μ_met,0_^–1^ + *aT*^3/2^ and hop between the clusters with a mobility μ_hop_ = μ_∞_exp(−*E*_A_/*kT*) to give an overall mobility μ_QPS_:
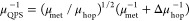
Here, Δ = (*c* – *c*_c_)/*c*_c_ (where *c* and *c*_c_ are the concentration and critical concentration of hopping links, respectively) measures the dimensionless distance to the metal–insulator transition (MIT). The epi-SLs discussed in this report contain highly-uniform regions of epitaxially-fused QDs interspersed with a variety of point defects such as missing necks and QD vacancies. It is thus plausible that holes delocalize within the highly-uniform regions of the epi-SL and then hop between these regions. The QPS model yields the best fits as quantified by the residual sum of squares (RSS) method (Figure 5b and Supporting Discussion 4). The small Δ of the two thinner, higher-mobility devices (Devices 1 and 2) suggests that the concentration of metallic clusters in these devices approaches the MIT to within a few percent (Table 1). Using the established critical exponent ν = 1.33,^36^ this implies a localization length of 10–20 QDs for these two devices. Alternatively, we found localization lengths of 10–15 nm using the mixed conduction model that Lan et al. recently employed to assess transport in HgTe QD thin films (Table 1 and Figure S28).^23^ Such localization lengths correspond to delocalization of holes within 3D clusters of 15–45 QDs.

Although the QPS model provides satisfactory fits of the μ(*T*) data with physically sensible values, the picture of transport within epi-SLs remains unclear. Short localization lengths indicate that carriers remain localized within small clusters of QDs, likely as a result of the disorder in epi-SL neck size (including missing necks) and electronic coupling.^8,13^ Surface/interface states, QD positional and orientational disorder, and point charges within the films can also disrupt the periodicity of the energy landscape and thereby localize charge carriers. Ongoing efforts to incorporate more perfect epi-SLs^13,14,37,12^ into smaller FETs (<0.5 μm channels) combined with strategies to eliminate or fill^38^ carrier traps should soon enable electrical measurements of individual delocalized domains within epi-SL grains. Such small devices will require particularly low-resistance electrical contacts. It is also important to eliminate the bias-stress effect in order to obtain unambiguous μ(*T*) data, which we have accomplished with amorphous PbSe QD films^39^ but not yet with epi-SLs. The process for fabricating single-grain epi-SL devices demonstrated here sets the stage for systematic FET- and Hall-based charge transport studies of emergent miniband physics in these materials.

## Supporting Information Available

Experimental methods, QD optical spectrum, structural and electrical characterization of each device, structure and transport data, transient correction methodology, effect of annealing, detailed μ(*T*) data for each device, transport modeling, device fabrication details, estimate of site energy disorder and charging energy, comparison of charge transport models, and quantification of μ(*T*) fits (PDF)
