# Supplementary material for: High-Mobility Hole Transport in Single-Grain PbSe Quantum Dot Superlattice Transistors
Source: Nano Lett. 2022 Nov 21;22(23):9578–85. doi: 10.1021/acs.nanolett.2c03657 (PMC9756332; doi:10.1021/acs.nanolett.2c03657)
Supplement: Supplementary file 1 — nl2c03657_si_001.pdf [file nl2c03657_si_001.pdf]

Supporting Information

for

High-mobility hole transport in single-grain PbSe quantum dot superlattice transistors

Alex Abelson,[1] Caroline Qian,[2] Zachary Crawford,[4] Gergely T. Zimanyi,[4] Matt Law[1,2,3]*

[1]*Department of Materials Science and Engineering, University of California, Irvine, Irvine, CA. 92697 USA*
[2]*Department of Chemical and Biomolecular Engineering, University of California, Irvine, Irvine, CA. 92697 USA*
[3]*Department of Chemistry, University of California, Irvine, Irvine, CA. 92697 USA*
[4]*Department of Physics, University of California, Davis, Davis, CA. 95616 USA*

Methods

Materials. All chemicals were used as received unless otherwise noted. Lead oxide (PbO, 99.999%) and selenium shot (99.999%) were purchased from Alfa Aesar. Oleic acid (OA, technical grade, 90%), diphenylphosphine (DPP, 98%), 1-octadecene (ODE, 90%), anhydrous ethylene glycol (EG, 99.8%), anhydrous acetonitrile (99.99%), anhydrous hexanes (99%), anhydrous toluene (99.8%), extra dry acetone (99.8%), acetone (>99.5 %) for substrate cleaning outside of the glovebox, 3-mercaptopropyltrimethoxysilane (3-MPTMS, 95%), sodium hydroxide (97%), glycine (99%), ammonium thiocyanate (99.99%), trimethylaluminum (97%), 1,2-ethanedithiol (>98%), hexamethyldisilazane (HMDS, 99.9%), anhydrous *p*-xylene (99%), isopropanol (>99.5 %), anhydrous tetrachloroethylene (TCE, 99%), and methyl isobutyl ketone (MIBK, 99%) were purchased from Sigma-Aldrich. Trioctylphosphine (technical grade, >90%) was acquired from Fluka and mixed with Se shot for 24 hr to form a 1 M trioctylphosphine-Se stock solution. Anhydrous 1,2-ethylenediamine (EDA, >98.0%) was purchased from TCI.

Microposit S1808 photoresist, Microposit MF-319 developer, and 950 A5 (PMMA) e-beam resist were purchased from Kayaku Advanced Materials. Gold shot (99.99%) was purchased from Plasmaterials. Chromium plated tungsten rods and molybdenum evaporation boats were purchased from Kurt Lesker. 18.2 MΩ water (Milli-Q Gradient) was used for substrate cleaning, atomic layer deposition (ALD), and alumina etching. For the latter two processes, the water was freeze-pump-thawed three times prior to use.

Quantum dot synthesis. PbSe QDs were synthesized and purified using standard air-free techniques. PbO (1.50 g), OA (5.00 g), and ODE (10.00 g) were mixed and degassed in a three-neck round-bottom flask at room temperature. Then the mixture was heated at 120 °C under vacuum to form lead oleate ($Pb(OA)_2$) and dry the solution. After 1 hour, the $Pb(OA)_2$ solution was heated to 180 °C under argon flow and 9.5 mL of a 1 M solution of TOP-Se containing 200 μL of DPP was rapidly injected into this hot solution. An immediate darkening of the solution was observed, and the QDs were grown for 105 seconds at ~150 °C. The reaction was quenched with a liquid nitrogen bath and injection of 10 mL of anhydrous hexanes. The QDs were purified in an N_2-filled glovebox (<0.5 ppm O_2) by adding 18 mL of acetonitrile and 6 mL of toluene to the reaction solution, collecting the QDs by centrifugation, performing three cycles of redispersion/precipitation using toluene/acetonitrile (3 mL/23 mL), and then drying under vacuum and storing the QDs as a powder in the glovebox.

Basic characterization. Optical absorbance measurements of QDs dispersed in TCE were performed with a PerkinElmer Lambda 950 spectrophotometer. Neat TCE served as a background. Scanning electron microscopy was performed with an FEI Magellan 400L XHR SEM operating at 10 kV and 100 pA. FTIR transmission spectra of SL films on double-side polished intrinsic Si substrates were acquired in dry air on a Nicolet 6700 spectrometer at a

resolution of 4 cm^{-1} with a blank Si substrate as the background. AFM was performed using an Asylum Research Jupiter XR Atomic Force Microscope.

Substrate fabrication. All single-grain field-effect transistors (FETs) were fabricated on p^{++} (100)-oriented Si wafers coated with a 200 nm thick dry thermal oxide layer (Addison Engineering). All substrate fabrication steps were performed in air. The wafers were cleaned by sequential rounds of sonication in acetone, isopropanol, and water, dried on a hotplate at 110 ºC for 15 minutes, plasma cleaned in a mixture of O_2 and Ar for 10 minutes, and then photopatterned using a custom photomask and metalized with Cr/Au to produce a square array of contact pads and fiducial markers (Fig. 1c). Photopatterning started with the deposition of a photoresist primer layer by spin coating a 20% volume solution of HMDS (hexamethyldisilazane) in *p*-xylene at 3500 rpm for 120 seconds. A layer of S1808 photoresist was then spin cast onto the HMDS-treated wafer at 3500 rpm for 120 seconds. The photoresist layer was soft-baked at 90 ºC for 30 minutes. Next, the photomask was aligned over the wafer and illuminated with an Ushio USH-508SA UV lamp for 2.6 seconds. The wafer was then soaked in photoresist developer (MF-319) for 60 seconds, followed by soaking and gentle rinsing with water. The wafer was then dried for 5 minutes at 110 ºC and O_2 plasma cleaned for 5 minutes. Chromium and gold were deposited in a thermal evaporation system inside of an N_2-filled glovebox (<0.5 ppm O_2). The base pressure of the evaporator was 2×10^{-6} mbar. All metal depositions consisted of 5 nm of Cr (@ 0.1-0.2 Å/s) and 45 nm of Au (@ 0.7 Å/s) and utilized a sample rotation stage (~10 rpm). Lift-off of the photoresist was performed by sonicating in acetone. The wafers were then diced by hand into 1.2 × 1.2 cm chips and individual chips were photopatterned as described above to produce a bare 550 × 550 μm square at the center of its array of contact pads. Next, the chips were O_2 plasma cleaned for 5 minutes and soaked in 100

mM MPTMS in toluene for 1 hour in the glovebox to improve epi-SL adhesion. The chips were then rinsed in neat toluene and blown dry prior to epi-SL deposition.

Single-grain epi-SL FET fabrication. SL fabrication was performed in N_2-filled gloveboxes with <0.5 ppm O_2. 60 μL of a 10 mg/mL (for Devices 1 and 2) or 19 mg/mL (for Devices 3-8) dispersion of PbSe QDs in hexanes was pipetted onto 7 mL of ethylene glycol in a Teflon well. After depositing the QD solution, the well was immediately covered by a glass plate. The hexane was allowed to slowly evaporate over 25-32 minutes, resulting in a smooth, dry oleate-capped QD film floating on the EG surface. 0.1 mL of 7.5 M ethylenediamine in acetonitrile was injected under an edge of the film and allowed to react for 20-30 seconds (shorter for the thinner films). The resulting epi-SL film was stamped onto the MPTMS-treated chip described in the previous section and the chip was rinsed with two rounds of acetone to remove the photoresist layer, followed by a rinse in acetonitrile. This procedure resulted in epi-SL film deposited only in the 550 × 550 μm square at the center of the chip. The chip was then transferred air-free to an in-glovebox ALD system and coated with 11 nm of amorphous alumina using trimethylaluminum and water at a substrate temperature of 60 ºC. Precursors were introduced to the ALD chamber using computer-controlled diaphragm valves in-line with a 130 sccm stream of N_2 carrier gas. Pulse and purge times were 20 ms and 55 seconds, respectively, for both precursors. The ALD-coated samples were imaged by SEM to locate epi-SL grains of interest and subsequently coated with an additional 22 nm of alumina (for a total of 33 nm) to ensure their long-term environmental stability. If needed, the chip was rinsed with water and IPA, then blown dry. Electrical contacts to the single grains were made by multiple rounds of electron beam lithography, alumina etching, and metallization. First, alumina was removed from the contact pads by photopatterning (using the S1808 resist process described above, including a 30-minute

dwell at 90 °C in ambient air) a protective resist layer (600 × 600 μm) over the 550 × 550 μm epi-SL film at the center of each chip and etching the alumina off the rest of the chip. Alumina etching was performed by immersing the chips in a pH 10 glycine-buffered aqueous NaOH solution that yields an etch rate of ~0.6 nm/min. After alumina etching, the chips were rinsed with water, acetone, water, and IPA, then blown dry. The acetone rinse removes the protective photoresist on the epi-SL film. Next, the chips were coated with PMMA e-beam resist by spin coating at 2750 rpm for 60 seconds, then baked on a hotplate in air at 150 ºC for 3 minutes. Chips were transferred to the SEM and coarse electrodes written from the contact pads to locations within several μm of each grain of interest using a Nanometer Pattern Generation System (NPGS) at 30 keV and a total electron dose of 550 $\mu C/cm^2$. The chips were then removed from the SEM and developed in air for 40 seconds in a 1:3 mixture of MIBK and IPA, rinsed with neat IPA, and blown dry. Following metallization (identical to the procedure above), lift-off was performed using 3-6 hour soaks in acetone in the glovebox. Each sample was then rinsed vigorously in acetone and acetonitrile and blown dry. PMMA was spin coated onto the chip at 2750 rpm for 60 seconds and baked on a hotplate at 150 ºC for 3 minutes in air. The chip was transferred back into the SEM for EBL patterning of fine electrodes that connect the coarse electrodes with the edges of each grain and define source and drain contacts of the FETs. The chips were then removed from the SEM, developed in air for 40 seconds in a 1:3 mixture of MIBK and IPA, rinsed with neat IPA, blown dry, transferred back into the glovebox, and etched for 55 minutes to remove the alumina in the fine electrode areas. After etching, the chips were rinsed in the glovebox with degassed water and blown dry with N_2. The chips were loaded again air-free into the thermal evaporator and metalized with Cr/Au (identical to the procedure above). Lift-off was performed using 3-6 hour soaks in acetone in the glovebox. Each sample was then

rinsed vigorously in acetone and acetonitrile and blown dry. Finally, an additional 11 or 22 nm of alumina was deposited by ALD to further guarantee the air stability of the devices (44-55 nm of alumina in total).

Electrical characterization. All electrical measurements were performed in the dark using a Keithley 2636B source-measure unit controlled by homebuilt Labview software. Sample cooling was accomplished using an ARS DE-202S cryostat and Lakeshore 336 temperature controller with a calibrated DT-670 Si diode. The base pressure of the system was 8.6×10^{-8} Torr at 14 K (Pfeiffer HiCube 80 Eco turbopump). Single-grain FET chips were mounted on a thin sapphire window, which was in turn mounted to the copper cold finger of the cryostat. Apiezon N thermal grease was applied on all surfaces to ensure good thermal contact. The accuracy of temperature readings was verified by mounting the Si diode directly on a test chip and measuring the base temperature (11.5 K). This base temperature was identical to the base temperature achieved during a normal measurement (with the Si diode adjacent to the chip), indicating that the chip and the Si diode were in thermal equilibrium. Linear carrier mobilities were calculated at $|V_G|$ = 45 V using the gradual channel approximation equation for transconductance:

$$\left.\frac{dI_{\mathrm{D}}}{dV_{\mathrm{G}}}\right|_{V_{\mathrm{SD}}} = \frac{W C_{\mathrm{ox}} V_{\mathrm{SD}}}{L} \mu_{\mathrm{lin}}$$

where C_{ox} = 17.5 nF cm^{-2} and W and L are the channel width and length, respectively.

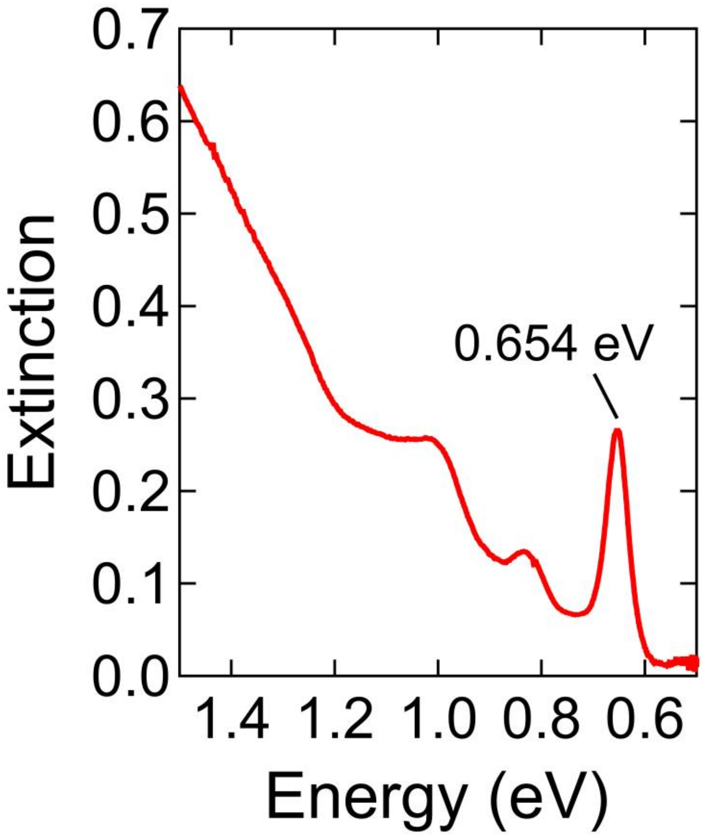

Figure S1. Optical extinction spectrum of 6.9 nm oleate-capped PbSe QDs dispersed in TCE. The first exciton appears at 0.654 eV, corresponding to an average QD diameter of 6.9 nm.[1] Neat TCE served as the background.

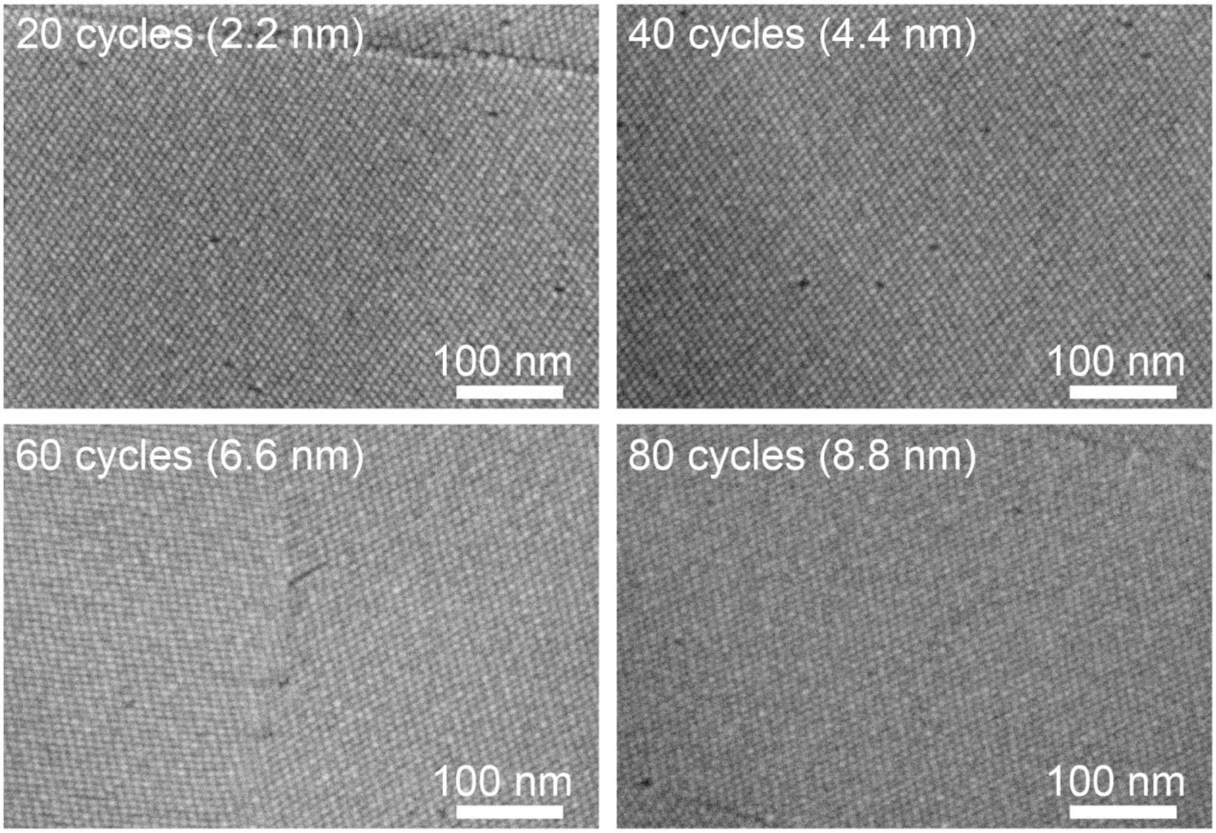

Figure S2. Electron transparency of the alumina encapsulation layer as a function of layer thickness. Alumina deposited by ALD was used to encapsulate the epi-SLs prior to SEM imaging. The growth rate of alumina at 60 ºC was determined to be 1.1 Å/cycle. SEM imaging of epi-SLs coated with varying thicknesses of alumina show that the QDs are visible through the alumina layer at alumina thicknesses up to and above 8.8 nm. As described in the text, imaging of the epi-SL grains was performed through an 11 nm thick layer of alumina.

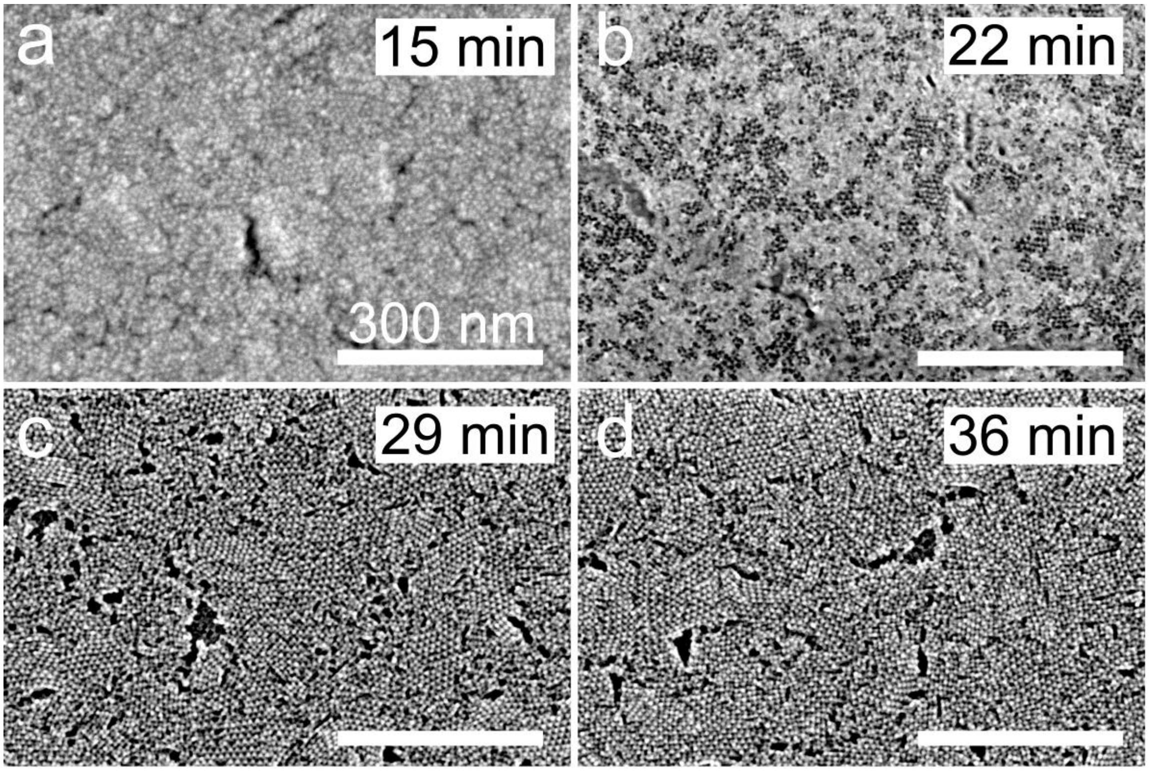

Figure S3. Wet etching of the ALD alumina layer. SEM image time series showing the removal of a 16.5 nm thick alumina overcoat on a spin-cast, amorphous QD film (not an epi-SL) in a pH 10 solution of glycine-buffered NaOH (see Methods). a) After 15 minutes of etching, the QDs remain encased in alumina. b) After 22 minutes, a discontinuous lacey alumina residue remains. c) After 29 minutes, the alumina layer has been completely removed, exposing the entire QD film. d) After 36 minutes, there are no noticeable morphological changes to the QDs, indicating that the etch solution does not rapidly degrade the QDs. All scale bars are 300 nm.

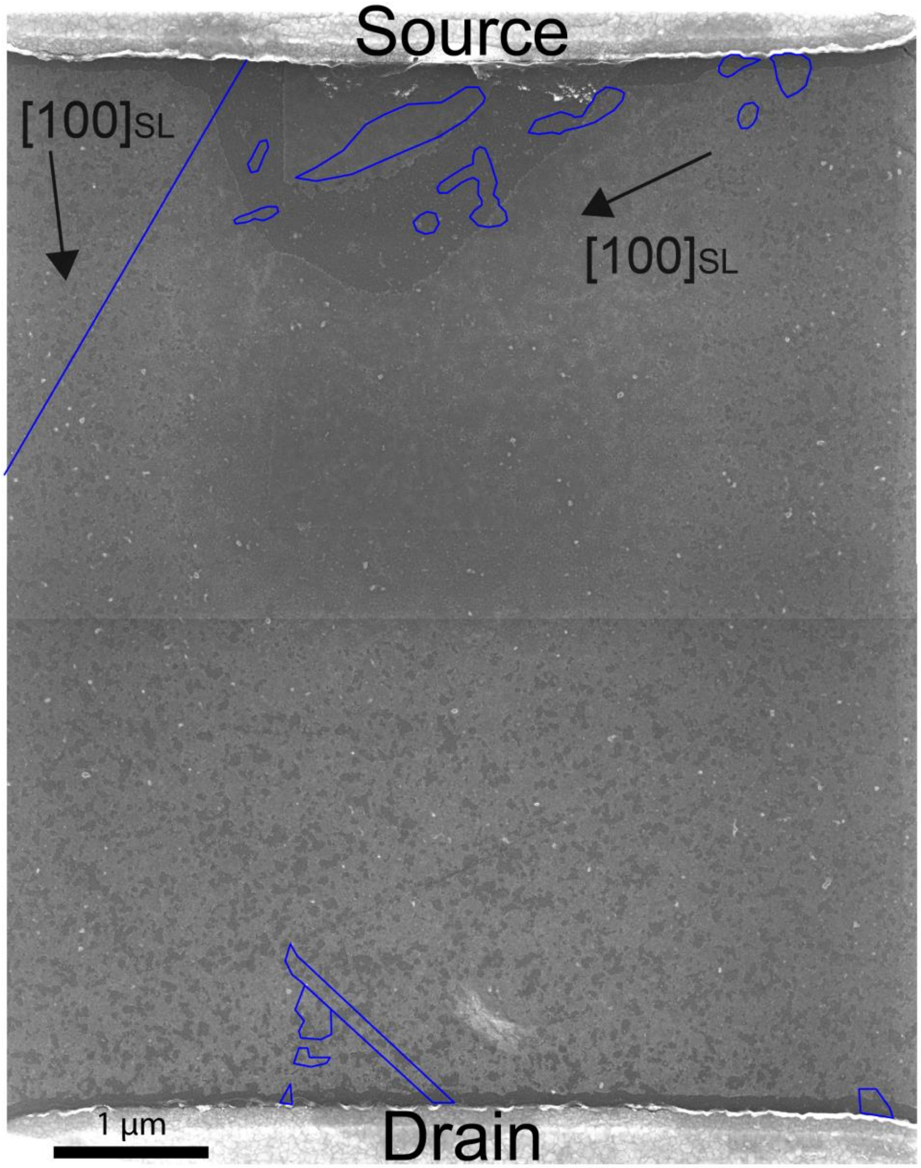

Figure S4. Postmortem SEM microstructural map of Device 1. This FET has a channel with dimensions of L = 6.8 µm and W = 5.9 µm. Blue lines denote twin planes. Most of the channel is spanned by a single $(01\bar{1})_{SL}$-oriented epi-SL grain (monocrystal) that contains 14 small twinned inclusions (outlined in blue). There is also a long diagonal twin plane in the upper left of the image that a small fraction of the carriers likely cross to transit the channel. Arrows denote the $[100]_{SL}$ directions in the major grain and the minor grain. The angles between the transport direction and the $[100]_{SL}$ directions of the major and minor grains are 64º and 8º, respectively. FFT analysis of ten areas in the channel shows that the inter-QD distance along $[100]_{SL}$ (i.e., the lattice constant) is 6.86 ± 0.11 nm. The area per QD is 57.5 ± 0.8 nm^2/QD. The film thickness in the channel was estimated by AFM to be 35 nm (see Table S1). Residue in the channel is a combination of residual (un-etched) alumina and carbon accumulation during imaging. The faint horizontal line (seam) in the middle of the image is an image stitching artifact (the image is a mosaic of two SEM images).

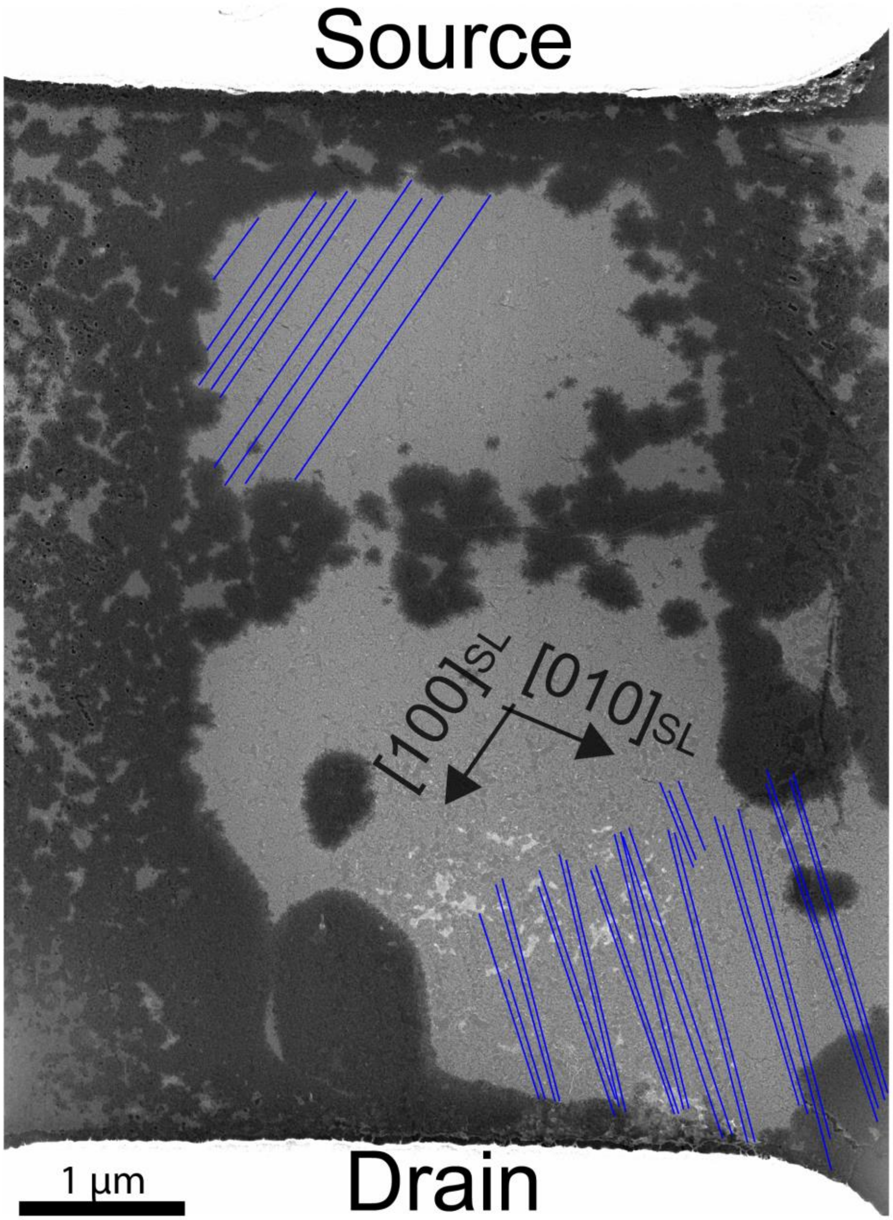

Figure S5. Postmortem SEM microstructural map of Device 2. This FET has a channel with dimensions of L = 6.4 µm and W = 5.1 µm. Blue lines denote twin planes. Most of the channel is spanned by a single $(100)_{SL}$-oriented epi-SL grain (monocrystal) that contains small twinned inclusions (outlined in blue). Arrows denote the $[100]_{SL}$ and $[010]_{SL}$ directions in the grain. The angles between the transport direction and the $[100]_{SL}$ directions of the major grain is 35˚. This particular device was exposed to an extremely long SEM exposure after electrical characterization, which led to a thick buildup of carbon. The film thickness in the channel was measured by AFM to be 35.2 ± 1.3 nm. Removal of this layer required successive rounds of O_2 plasma cleaning and alumina etching which caused visible degradation of the QDs. The QD-QD spacing and QD density are therefore too unreliable to report.

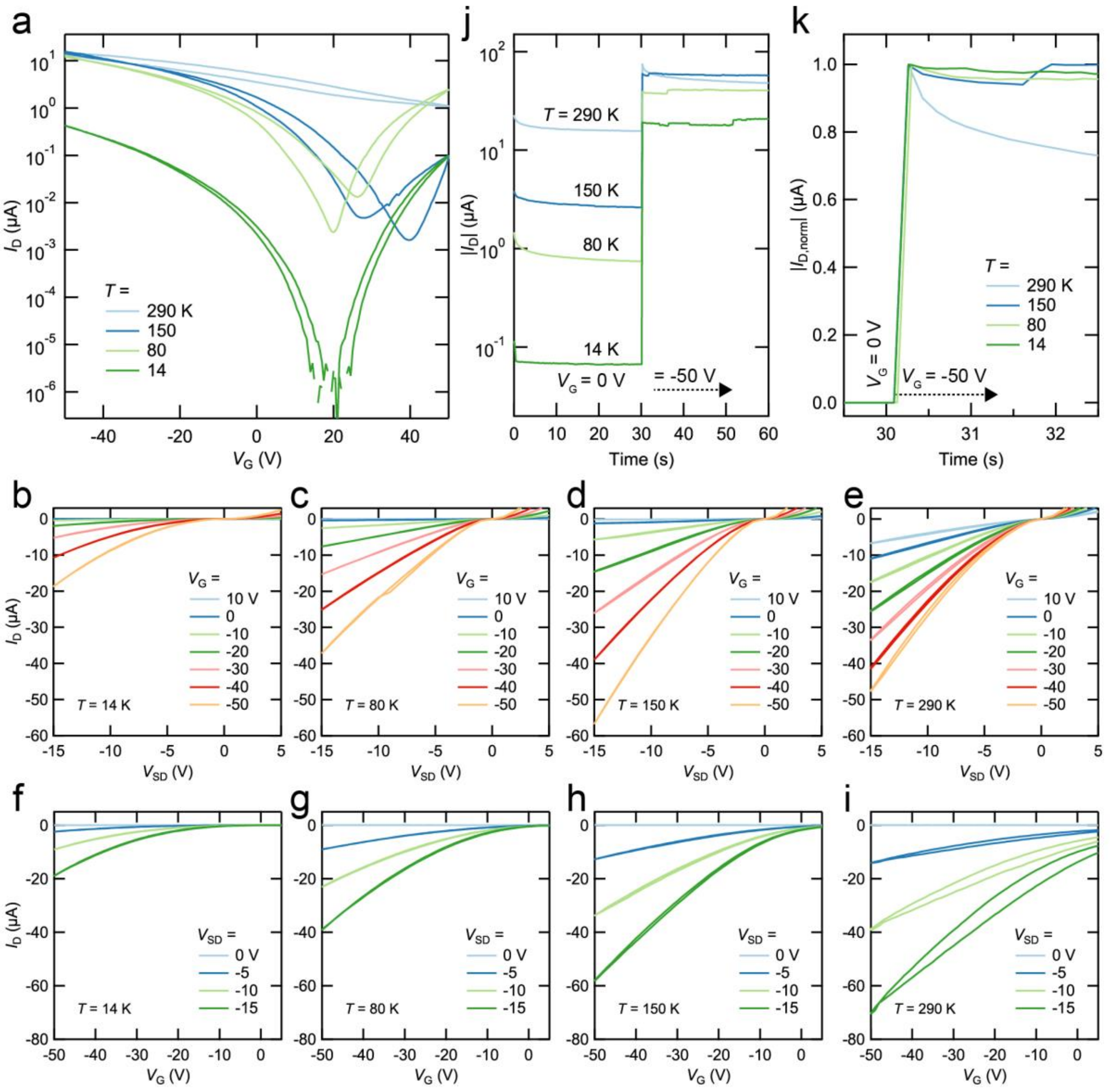

Figure S6. Electrical data for Device 2. (a) Full-sweep (V_G = ±50 V) transfer curves acquired at T = 14, 80, 150, and 290 K using V_{SD} = 2 V and a sweep rate of 5 V/s. The device is *p*-channel at room temperature and becomes increasingly ambipolar at lower temperature. I_{OFF} drops dramatically with decreasing temperature. (b-e) Output curves acquired at T = 14, 80, 150, and 290 K, respectively, using a series of V_G values and a V_{SD} sweep rate of 5 V/s. V_{SD} was swept from 5 V to -15 V and then back to 5 V. (f-i) Corresponding transfer curves acquired at a series of V_{SD} values using a sweep rate of 200 V/s. V_{SD} was swept from 10 V to -50 V, then back to 10 V. (j) Time traces of $|I_D|$ as V_G is stepped from 0 V to -50 V. Here, V_{SD} = -15 V. (k) Comparison of the normalized $|I_D|$ time traces from (j), with $I_{\mathrm{D,norm}} = \frac{I(t) - I_{V_{\mathrm{G}}=0}}{I_{\mathrm{peak}} - I_{V_{\mathrm{G}}=0}}$, where $I(t)$ is the drain current at time t, $I_{V_{\mathrm{G}}=0}$ is the drain current at V_G = 0 V, and I_{peak} is the peak drain current.

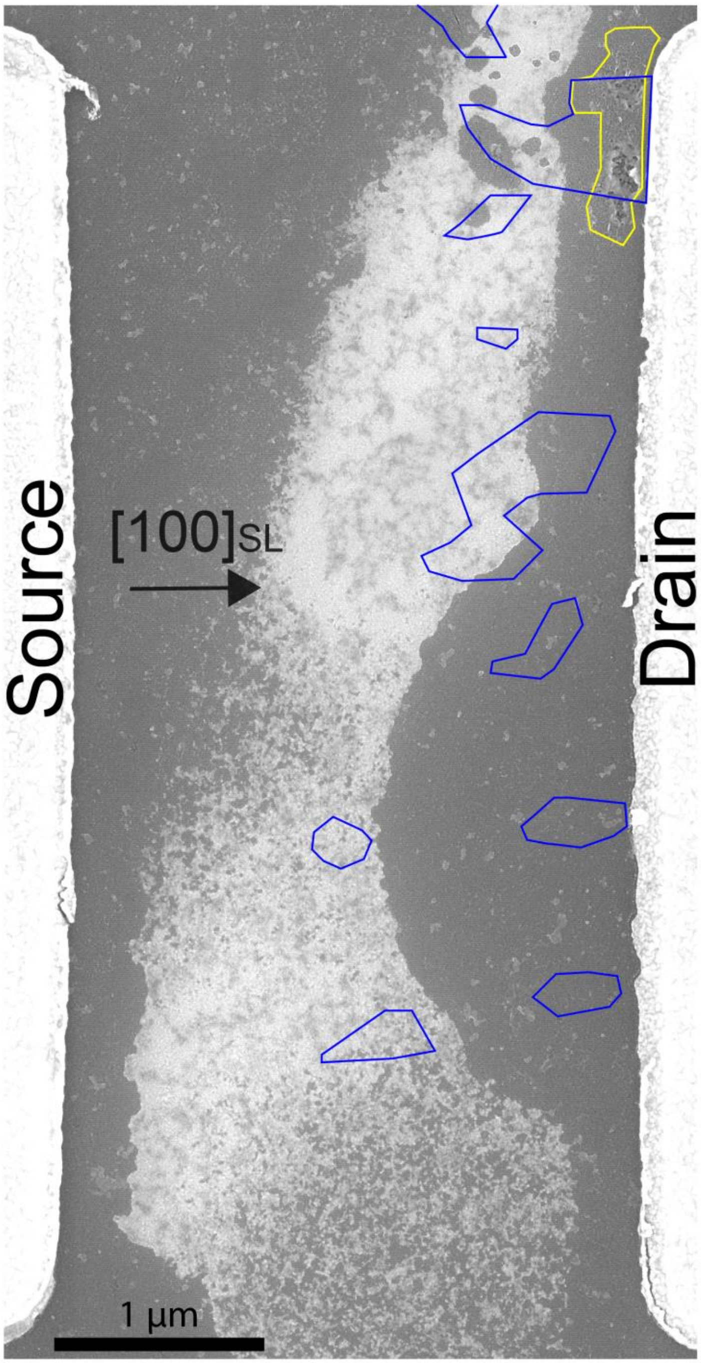

Figure S7. Postmortem SEM microstructural map of Device 3. This FET has a channel with dimensions of L = 3.1 um and W = 6.8 um. Blue lines denote twin planes. Yellow lines enclose a highly-defective region. The channel is spanned by a single $(01\bar{1})_{SL}$-oriented epi-SL grain that contains 10 twinned inclusions (outlined in blue). The $[100]_{SL}$ direction (arrow) is parallel to the transport direction. FFT analysis of seven areas in the channel shows that the inter-QD distance along $[100]_{SL}$ (i.e., the lattice constant) is 6.81 ± 0.05 nm. The area per QD is 60.4 ± 0.5 nm^2/QD. The film thickness in the channel was measured by AFM to be 77.5 ± 10.3 nm. Residue in the channel is incompletely-etched alumina.

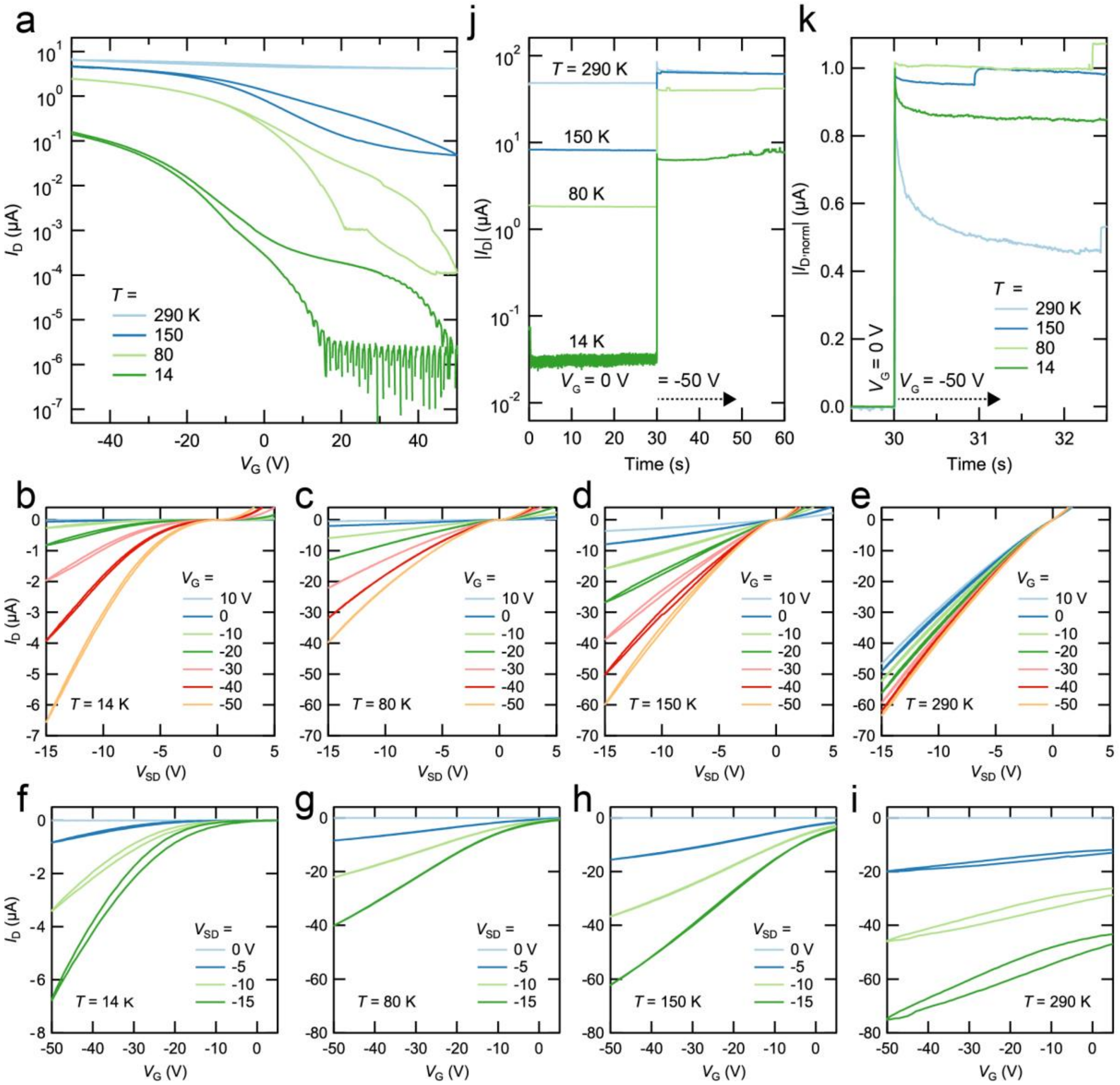

Figure S8. Electrical data for Device 3. (a) Full-sweep ($V_G = \pm 50$ V) transfer curves acquired at T = 14, 80, 150, and 290 K using V_{SD} = 2 V and a sweep rate of 5 V/s. The device is *p*-channel over the entire range of temperature and gate bias. I_{OFF} drops dramatically with decreasing temperature. (b-e) Output curves acquired at T = 14, 80, 150, and 290 K, respectively, using a series of V_G values and a V_{SD} sweep rate of 5 V/s. V_{SD} was swept from 5 V to -15 V and then back to 5 V. (f-i) Corresponding transfer curves acquired at a series of V_{SD} values using a sweep rate of 200 V/s. V_{SD} was swept from 10 V to -50 V, then back to 10 V. (j) Time traces of $|I_D|$ as V_G is stepped from 0 V to -50 V. Here, V_{SD} = -15 V. (k) Comparison of the normalized $|I_D|$ time traces from (j), with $I_{\mathrm{D,norm}} = \frac{I(t) - I_{V_{\mathrm{G}}=0}}{I_{\mathrm{peak}} - I_{V_{\mathrm{G}}=0}}$, where $I(t)$ is the drain current at time t, $I_{V_{\mathrm{G}}=0}$ is the drain current at V_G = 0 V, and I_{peak} is the peak drain current.

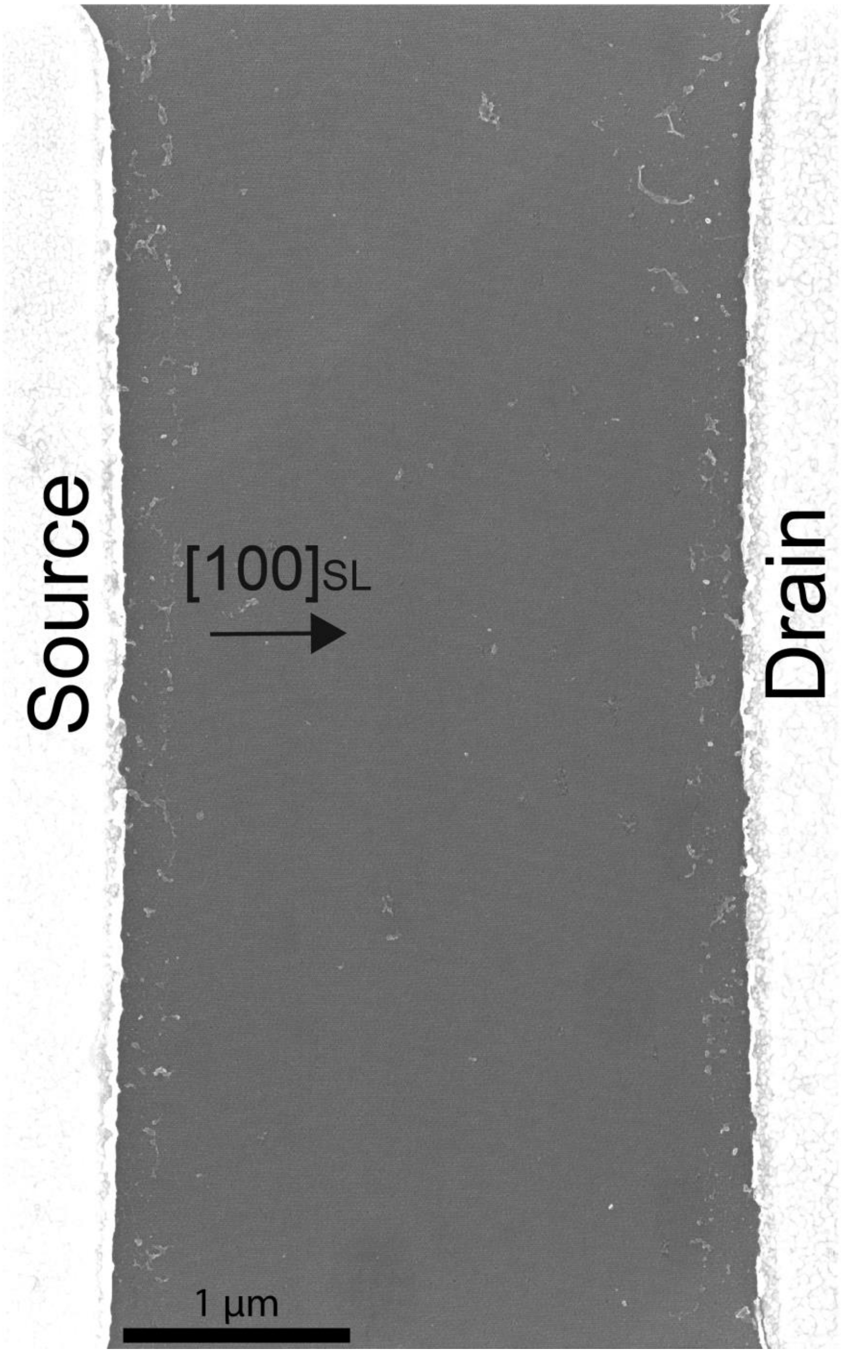

Figure S9. Postmortem SEM microstructural map of Device 4. This FET has a channel with dimensions of L = 3.1 µm and W = 6.6 µm. The channel is spanned by a single $(01\bar{1})_{SL}$-oriented epi-SL grain (monocrystal). There are no twin planes or other grain boundaries present in the channel (no inclusions). The $[100]_{SL}$ direction (arrow) is parallel to the transport direction. FFT analysis of ten areas in the channel shows that the inter-QD distance along $[100]_{SL}$ (i.e., the lattice constant) is 7.08 ± 0.11 nm. The area per QD is 62.6 ± 0.9 nm^2/QD. The film thickness in the channel was measured by AFM to be 84.5 ± 5.0 nm. The residue in the channel is a mixture of residual alumina and carbon deposited during imaging.

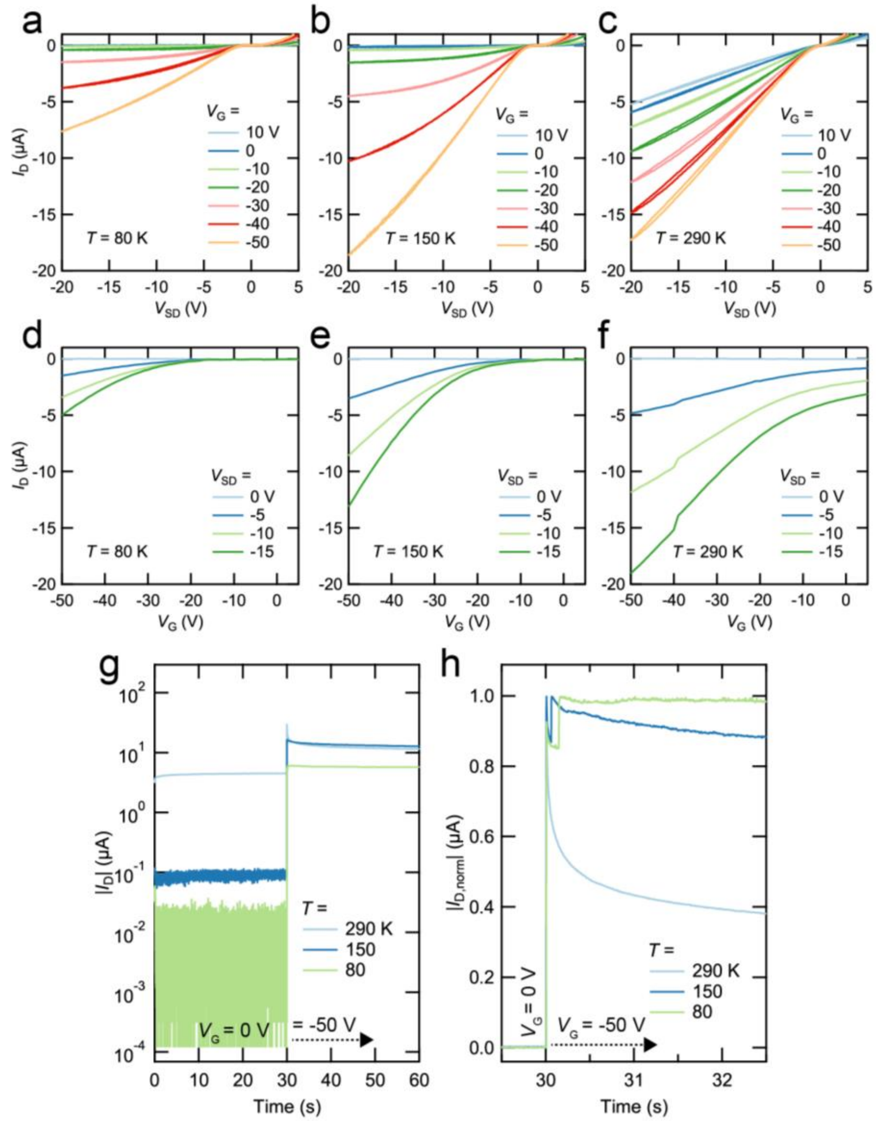

Figure S10. Electrical data for Device 4. (a-c) Output curves acquired at T = 80, 150, and 290 K, respectively, using a series of V_G values and a V_{SD} sweep rate of 22 V/s. V_{SD} was swept from 5 V to -15 V, then back to 5 V. (d-f) Corresponding transfer curves acquired at a series of V_{SD} values using a sweep rate of 200 V/s. V_G was swept from 10 V to -50 V. (g) Time traces of $|I_D|$ as V_G is stepped from 0 V to -50 V. Here, V_{SD} = -15 V. (h) Comparison of the normalized $|I_D|$ time traces from (g), with $I_{D,\mathrm{norm}} = \frac{I(t)-I_{V_G=0}}{I_{\mathrm{peak}}-I_{V_G=0}}$, where $I(t)$ is the drain current at time t, $I_{V_G=0}$ is the drain current at V_G = 0 V, and I_{peak} is the peak drain current.

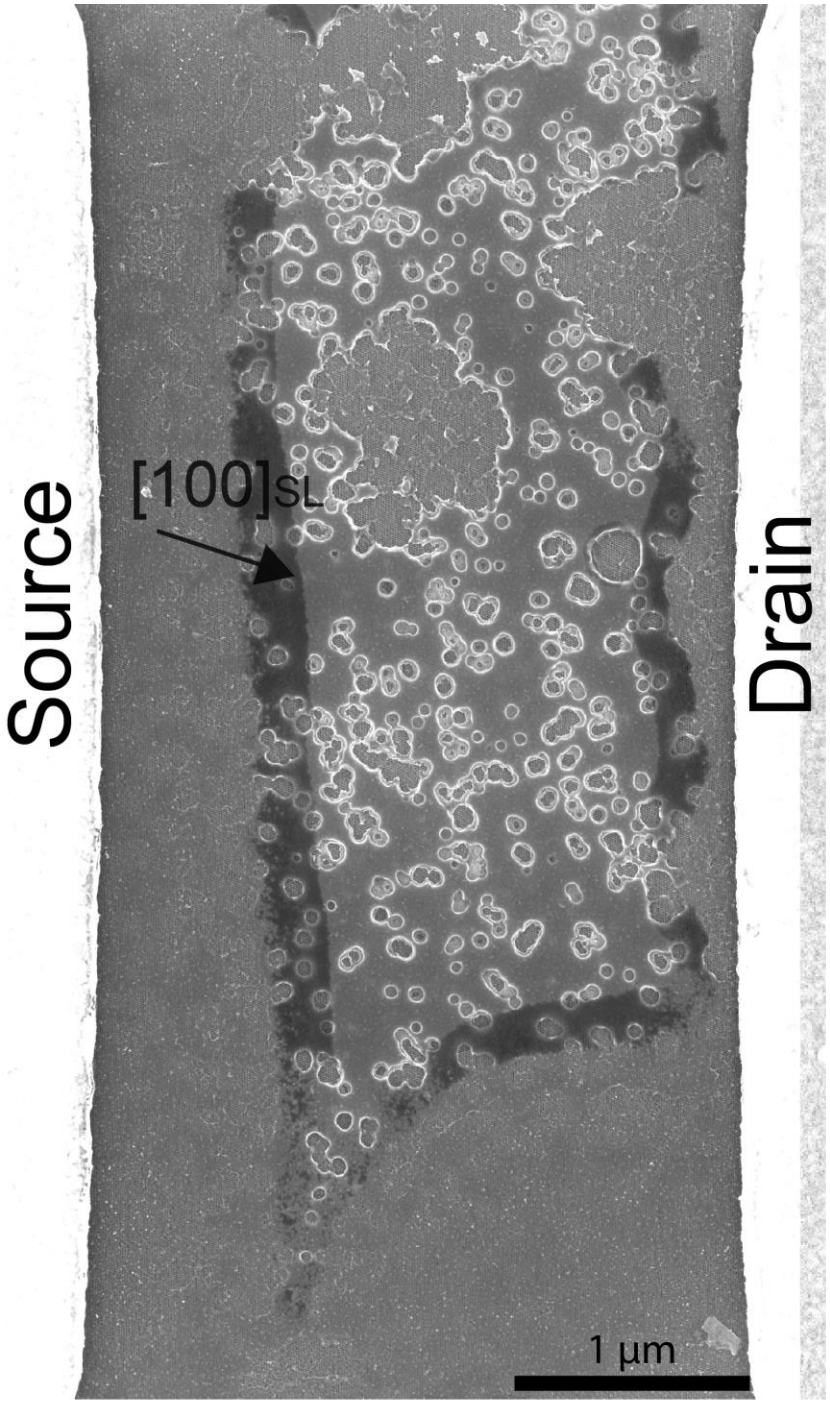

Figure S11. Postmortem SEM microstructural map of Device 5. This FET has a channel with dimensions of L = 2.7 µm and W = 5.9 µm. The channel is spanned by a single $(01\bar{1})_{SL}$-oriented epi-SL grain (monocrystal). There are no twin planes or other grain boundaries present in the channel (no inclusions). The $[100]_{SL}$ direction (arrow) is 18° from the transport direction. FFT analysis of six areas in the channel shows that the inter-QD distance along $[100]_{SL}$ (i.e., the lattice constant) is 7.03 ± 0.03 nm. The area per QD is 61.0 ± 0.4 nm^2/QD. The film thickness in the channel was measured by AFM to be 57.5 ± 7.0 nm. The residue in the channel is a mixture of residual alumina and carbon deposited during imaging.

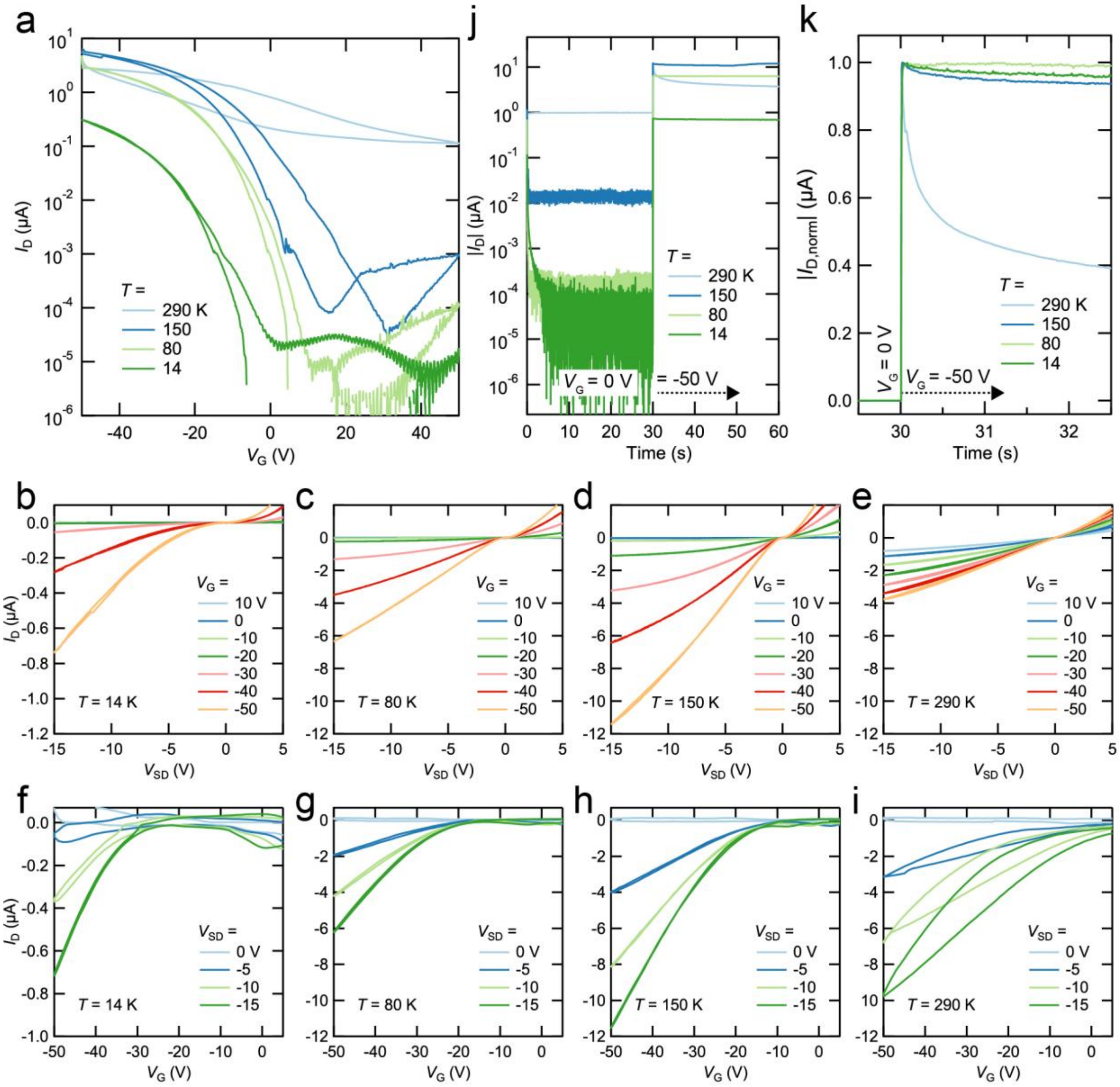

Figure S12. Electrical data for Device 5. (a) Full-sweep (V_G = ±50 V) transfer curves acquired at T = 14, 80, 150, and 290 K using V_{SD} = 6 V and a variable sweep rate. The device is *p*-channel at T = 290 K and begins to show ambipolar transport at lower temperature. I_{OFF} drops dramatically with decreasing temperature. (b-e) Output curves acquired at T = 14, 80, 150, and 290 K, respectively, using a series of V_G values and a V_{SD} sweep rate of 5 V/s. V_{SD} was swept from 5 V to -15 V and then back to 5 V. (f-i) Corresponding transfer curves acquired at a series of V_{SD} values using a sweep rate of 200 V/s. V_{SD} was swept from 10 V to -50 V, then back to 10 V. (j) Time traces of $|I_D|$ as V_G is stepped from 0 V to -50 V. Here, V_{SD} = -15 V. (k) Comparison of the normalized $|I_D|$ time traces from (j), with $I_{\mathrm{D,norm}} = \frac{I(t)-I_{V_{\mathrm{G}}=0}}{I_{\mathrm{peak}}-I_{V_{\mathrm{G}}=0}}$, where $I(t)$ is the drain current at time t, $I_{V_{\mathrm{G}}=0}$ is the drain current at V_G = 0 V, and I_{peak} is the peak drain current.

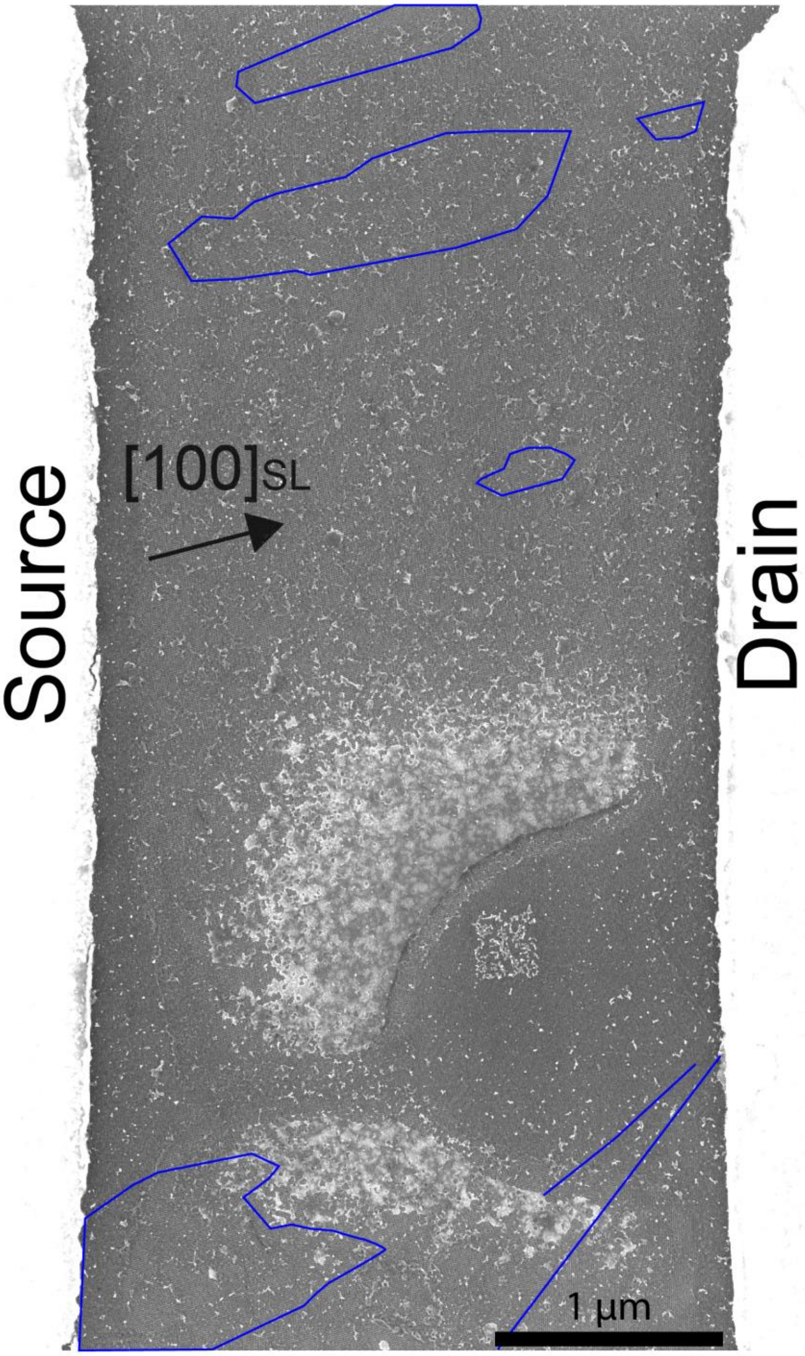

Figure S13. Postmortem SEM microstructural map of Device 6. This FET has a channel with dimensions of L = 2.8 μm and W = 5.8 μm. Blue lines denote twin planes. The channel is spanned by a single $(01\bar{1})_{SL}$-oriented epi-SL grain with several large twinned inclusions. The $[100]_{SL}$ direction (arrow) is 16º from the transport direction. FFT analysis of eight areas in the channel shows that the inter-QD distance along $[100]_{SL}$ (i.e., the lattice constant) is 6.92 ± 0.11 nm. The area per QD is 59.9 ± 0.5 nm^2/QD. The film thickness in the channel was measured by AFM to be 59.4 ± 6.0 nm. The residue in the channel is a mixture of residual alumina and carbon deposited during imaging.

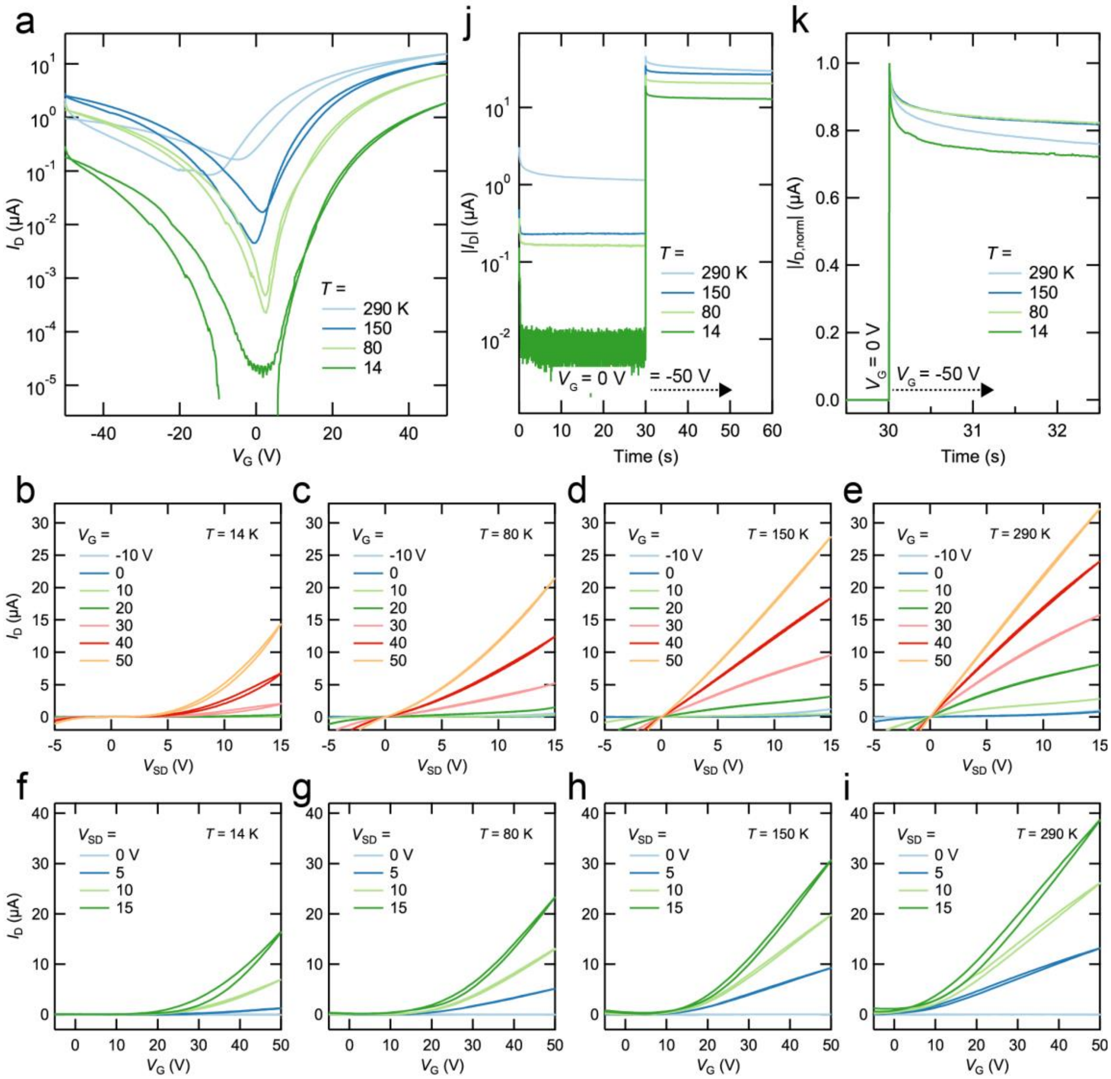

Figure S14. Electrical data for Device 6. (a) Full-sweep (V_G = ±50 V) transfer curves acquired at T = 14, 80, 150, and 290 K using V_{SD} = 2 V and a sweep rate of 5 V/s. The device is predominantly *n*-channel at room temperature because it was exposed to the electron beam for significantly more time than the other seven devices, which caused the shift in its polarity. It becomes increasingly ambipolar at lower temperature. I_{OFF} drops dramatically with decreasing temperature. (b-e) Output curves acquired at T = 14, 80, 150, and 290 K, respectively, using a series of V_G values and a V_{SD} sweep rate of 5 V/s. V_{SD} was swept from -5 V to 15 V and then back to -5 V. (f-i) Corresponding transfer curves acquired at a series of V_{SD} values using a sweep rate of 200 V/s. V_{SD} was swept from -10 V to 50 V, then back to -10 V. (j) Time traces of $|I_D|$ as V_G is stepped from 0 V to 50 V. Here, V_{SD} = 15 V. (k) Comparison of the normalized $|I_D|$ time traces from (j), with $I_{\mathrm{D,norm}} = \frac{I(t) - I_{V_{\mathrm{G}}=0}}{I_{\mathrm{peak}} - I_{V_{\mathrm{G}}=0}}$, where $I(t)$ is the drain current at time t, $I_{V_{\mathrm{G}}=0}$ is the drain current at V_G = 0 V, and I_{peak} is the peak drain current.

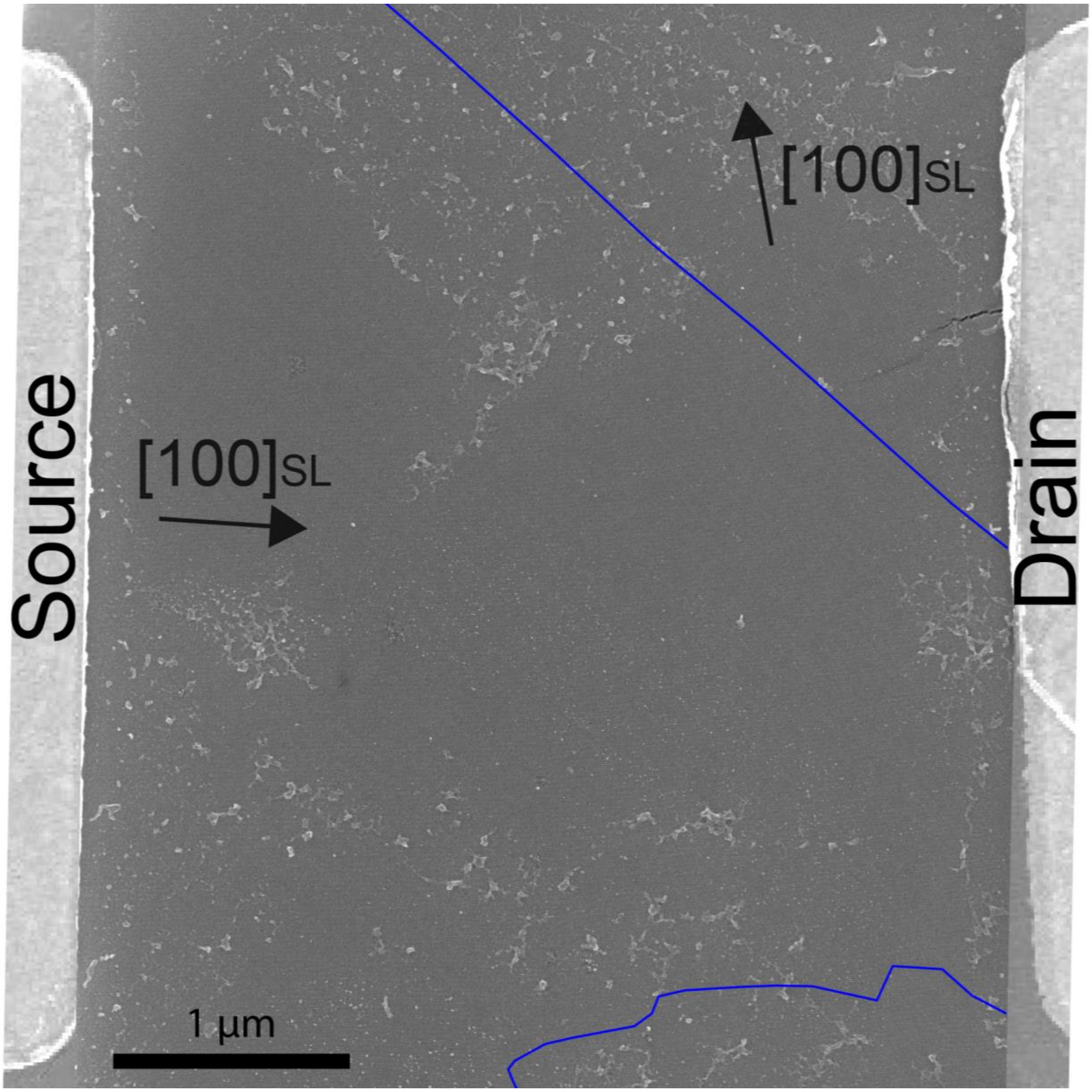

Figure S15. Postmortem SEM microstructural map of Device 7. This FET has a channel with dimensions of L = 4.4 μm and W = 4.6 μm. Blue lines denote twin planes. Most of the channel is spanned by a single $(01\bar{1})_{SL}$-oriented epi-SL grain (monocrystal) that is free of inclusions. There is also a long diagonal twin plane in the right half of the image that a large fraction of the carriers may cross to transit the channel. Arrows denote the $[100]_{SL}$ directions in the major grain and the minor grain. The angles between the transport direction and the $[100]_{SL}$ directions of the major and minor grains are 4º and 79º, respectively. FFT analysis of nine areas in the channel shows that the inter-QD distance along $[100]_{SL}$ (i.e., the lattice constant) is 7.06 ± 0.10 nm. The area per QD is 62.6 ± 1.1 nm^2/QD. The film thickness in the channel was measured by AFM to be 84.5 ± 4.7 nm. The residue in the channel is a mixture of residual alumina and carbon deposited during imaging.

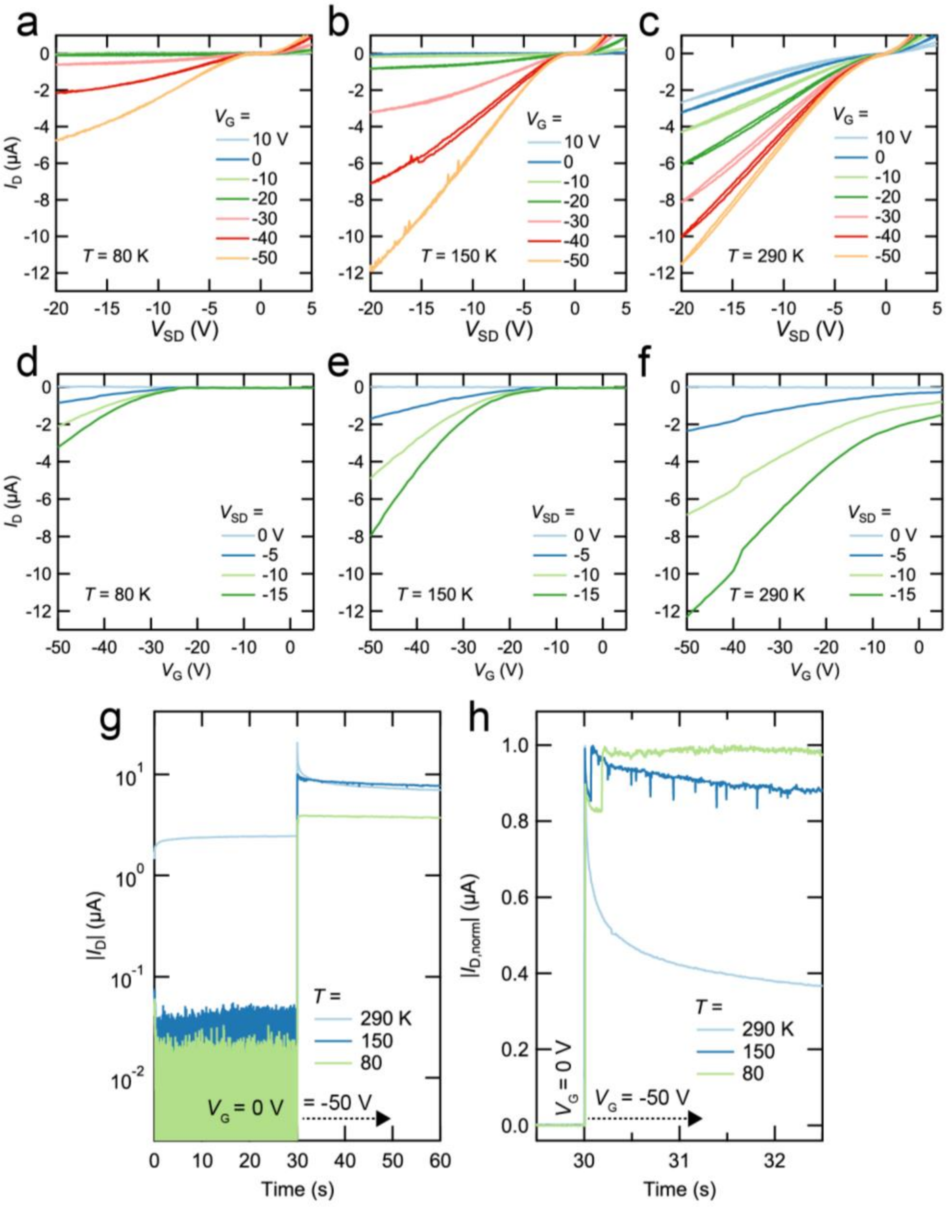

Figure S16. Electrical data for Device 7. (a-c) Output curves acquired at T = 80, 150, and 290 K, respectively, using a series of V_G values and a V_{SD} sweep rate of 22 V/s. V_{SD} was swept from 5 V to -15 V and then back to 5 V. (d-f) Corresponding transfer curves acquired at a series of V_{SD} values using a sweep rate of 200 V/s. V_G was swept from 10 to -50 V. (g) Time traces of $|I_D|$ as V_G is stepped from 0 V to -50 V. Here, V_{SD} = -15 V. (h) Comparison of the normalized $|I_D|$ time traces from (g), with $I_{\mathrm{D,norm}} = \frac{I(t) - I_{V_G=0}}{I_{\mathrm{peak}} - I_{V_G=0}}$, where $I(t)$ is the drain current at time t, $I_{V_G=0}$ is the drain current at V_G = 0 V, and I_{peak} is the peak drain current.

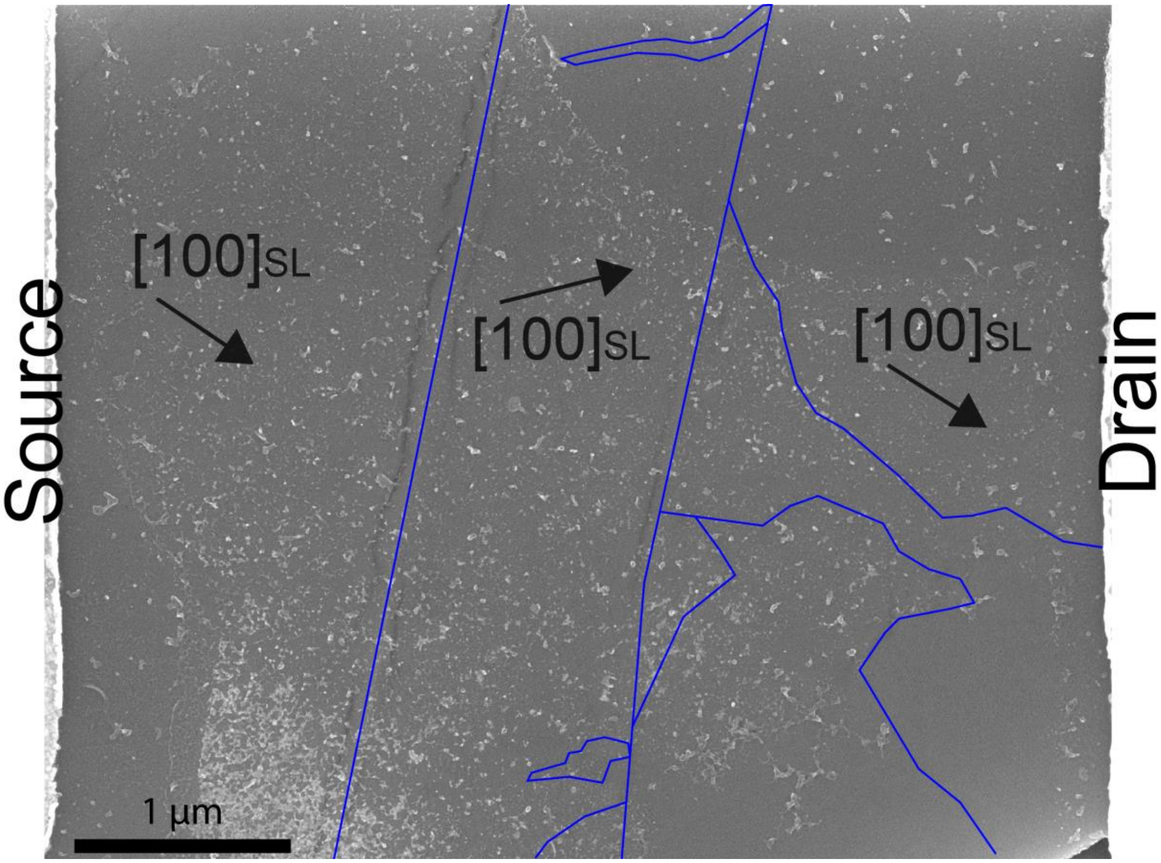

Figure S17. Postmortem SEM microstructural map of Device 8. This FET has a channel with dimensions of L = 4.4 µm and W = 5.5 µm. Blue lines denote twin planes. The channel consists of several $(01\bar{1})_{SL}$-oriented epi-SL grains (it is multi-crystalline) with several twin planes roughly perpendicular to the transport direction. Arrows denote the $[100]_{SL}$ directions in the three major grains. The angles between the transport direction and the $[100]_{SL}$ directions of the three grains are (left to right) 35º, 12º, and 32º. FFT analysis of seven areas in the channel shows that the inter-QD distance along $[100]_{SL}$ (i.e., the lattice constant) is 6.88 ± 0.05 nm. The area per QD is 60.9 ± 0.4 nm^2/QD. The film thickness in the channel was measured by AFM to be 82.5 ± 4.0 nm. The residue in the channel is a mixture of residual alumina and carbon deposited during imaging.

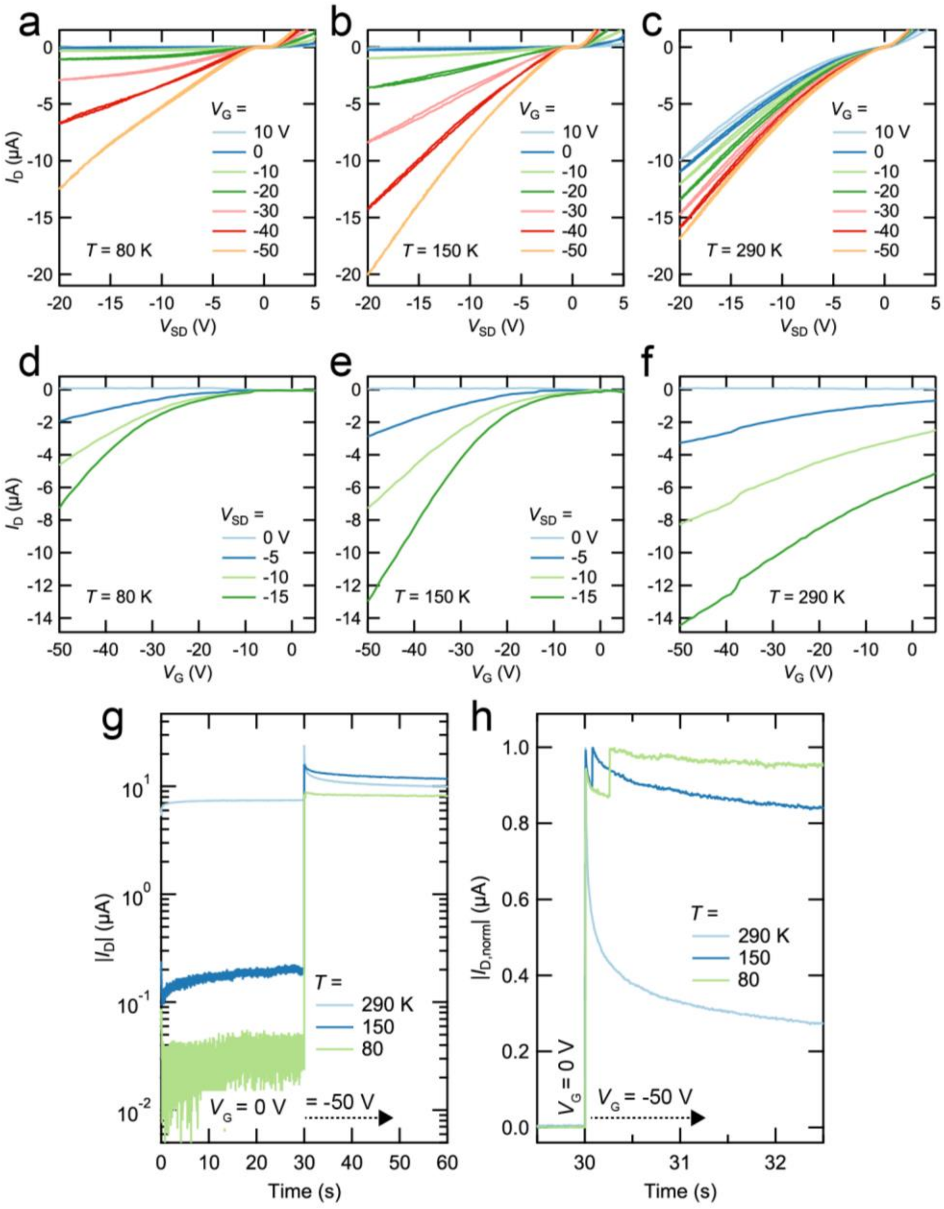

Figure S18. Electrical data for Device 8. (a-c) Output curves acquired at T = 80, 150, and 290 K, respectively, using a series of V_G values and a V_{SD} sweep rate of 22 V/s. V_{SD} was swept from 5 V to -15 V and then back to 5 V. (d-f) Corresponding transfer curves acquired at a series of V_{SD} values using a sweep rate of 200 V/s. V_G was swept from 10 to -50 V. (g) Time traces of $|I_D|$ as V_G is stepped from 0 V to -50 V. Here, V_{SD} = -15 V. (h) Comparison of the normalized $|I_D|$ time traces from (g), with $I_{\mathrm{D,norm}} = \frac{I(t)-I_{V_{\mathrm{G}}=0}}{I_{\mathrm{peak}}-I_{V_{\mathrm{G}}=0}}$, where $I(t)$ is the drain current at time t, $I_{V_{\mathrm{G}}=0}$ is the drain current at V_G = 0 V, and I_{peak} is the peak drain current.

Table S1. Structure and transport data for the single-grain epi-SL FETs.

	**Structural Parameters**						**μ_h (cm^2 V^{-1} s^{-1})^a (forward/reverse)**				**μ_e (cm^2 V^{-1} s^{-1})^b (reverse/forward)**			
Device	$L \times W$ (μm)	microstructure	$d_{[100]}$ (nm)^c	QDs/μm^2	ϕ (°)	thickness (nm)	T = 290 K	T = 150 K	T = 80 K	T = 14 K	T = 290 K	T = 150 K	T = 80 K	T = 14 K
1	6.8 ×<br>5.9	monocrystal w/ twin inclusions	6.86 ± 0.1	17,391 ±<br>242	64	35 ^{f}	6.7 ± 0.6<br>3.5 ± 0.5	6.8 ± 0.3<br>6.3 ± 0.4	4.2 ± 0.3<br>4.3 ± 0.4	0.11 ± 0.22<br>0.19 ± 0.01	-	0.53 ± 0.07 ^{e}<br>0.45 ± 0.01 ^{e}	1.7 ± 0.06 ^{e}<br>1.3 ± 0.03 ^{e}	0.57 ± 0.02^e<br>0.46 ± 0.02 ^{e}
2 ^{h}	6.4 ×<br>5.1	monocrystal w/ twin inclusions	7.0	20,000	35	35.2 ± 1.3	5.0 ± 0.3<br>4.4 ± 0.6	4.3 ± 0.4<br>3.7 ± 0.1	3.0 ± 0.4<br>3.1 ± 0.2	0.47 ± 0.26<br>0.40 ± 0.25	-	1.1 ± 0.1 ^{e}<br>0.81 ± 0.02 ^{e}	3.1 ± 0.1 ^{e}<br>2.5 ± 0.1 ^{e}	0.74 ± 0.04 ^{e}<br>0.62 ± 0.03 ^{e}
3	3.1 ×<br>6.8	monocrystal w/ twin inclusions	6.81 ± 0.05	16,556 ±<br>137	~0	77.5 ± 10.3	1.7 ± 0.6<br>1.0 ± 0.2	0.73 ± 0.06<br>0.58 ± 0.01	0.45 ± 0.01<br>0.40 ± 0.02	0.04 ± 0.04<br>0.03 ± 0.02	-	-	-	-
4	3.1 ×<br>6.6	monocrystal	7.08 ± 0.1	15,974 ±<br>230	~0	84.5 ± 5.0	-<br>0.60 ± 0.06	-<br>0.54 ± 0.11	-<br>0.29 ± 0.04	-	-	-	-	-
5	2.7 ×<br>5.9	monocrystal	7.03 ± 0.03	16,393 ±<br>108	18	57.5 ± 7.0	1.0 ± 0.2<br>0.72 ± 0.2	0.54 ± 0.05<br>0.59 ± 0.10	0.29 ± 0.03<br>0.34 ± 0.04	^{d}<br>^{d}	-	-	-	-
6	2.8 ×<br>5.8	monocrystal w/ twin inclusions	6.92 ± 0.1	16,694 ±<br>139	16	59.4 ± 6.0	-<br>0.18 ± 0.01 ^{e}	-<br>0.36 ± 0.01 ^{e}	-<br>0.14 ± 0.01 ^{e}	-<br>0.01 ± 0.01 ^{e}	1.7 ± 0.1<br>1.5 ± 0.1	1.4 ± 0.1<br>1.3 ± 0.1	0.69 ± 0.07<br>0.67 ± 0.01	^{d}<br>^{d}
7	4.4 ×<br>4.6	mostly monocrystal	7.06 ± 0.1	15,974 ±<br>281	4/79	84.5 ± 4.7	-<br>0.76 ± 0.07	-<br>0.36 ± 0.17	-<br>0.32 ± 0.07	-	-	-	-	-
8	4.4 ×<br>5.5	multi-crystal	6.88 ± 0.05	16,420 ±<br>108	35/12/<br>32	82.5 ± 4.0	-<br>1.2 ± 0.1	-<br>0.78 ± 0.01	-<br>0.39 ± 0.04	-	-	-	-	-

[a] Mobilities determined at V_G = -45 ± 3 V as $V_{SD} \rightarrow 0$ V (field-free), with transient correction (t = 225 ms for reverse sweep, t = 275 ms for forward sweep) unless otherwise noted.
[b] Mobilities determined at V_G = 45 ± 3 V as $V_{SD} \rightarrow 0$ V (field-free), with transient correction (t = 225 ms for forward sweep, t = 275 ms for reverse sweep) unless otherwise noted.
[c] $d_{[100]}$ is the center-to-center distance between QDs along $[100]_{SL}$, not the $(100)_{SL}$ d-spacing. Errors are standard deviations of multiple FFT fits of each image.
[d] Field correction of the mobility led to negative or otherwise unreliable mobility values and are therefore omitted.
[e] No transient or field correction was performed. Mobilities were calculated at V_{SD} = ±5 V and V_G = 45 ± 3.
[f] A reliable thickness measurement inside the channel was not possible. The thickness was assumed to be 35 nm based on the measured thickness of the surrounding epi-SL film.
[h] Device 2 is a $(100)_{SL}$-oriented epi-SL grain. All other devices are $(01\bar{1})_{SL}$-oriented grains.

Comparing the mobility values of Devices 1-8 reveals several noteworthy features of the data. First, the thinnest devices (Devices 1 and 2) have the highest mobility, probably because the topmost layers of the floating films (which become the bottommost layers of the FETs upon stamp transfer) are more fused in the thinner films and have thicker necks that provide better electronic coupling. Second, there is no obvious correlation between mobility and the rotational alignment of the SL relative to the FET channel. For example, Devices 4 and 5 have the same mobility despite their different values of ϕ (0° and 18°; Table S1). Third, the multi-crystalline control (Device 8) has a similar mobility to the monocrystalline devices of similar thickness, suggesting that intra-grain defects rather than grain boundaries limit transport, at least in these thicker devices. More samples must be measured to confirm and explain these initial findings.

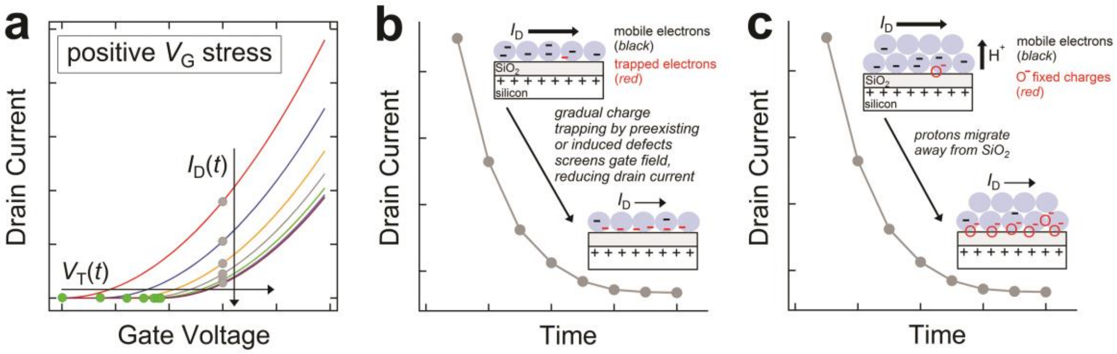

Figure S19. The bias-stress effect in QD FETs. (a) Schematic time evolution of the transfer (I_D-V_G) plot of an *n*-channel FET suffering from the bias-stress effect. Upon application of constant positive gate bias, a progressive build-up of immobile charges near the dielectric/QD interface screens the gate field, increasing V_T (green dots) and decreasing the drain current for a fixed set of bias conditions (e.g., grey dots). Cartoons of possible (b) electronic and (c) ionic mechanisms of the bias-stress effect. The drain current shows a quasi-exponential decay as trapped charges and/or drifting ions (e.g., protons) screen the gate field.

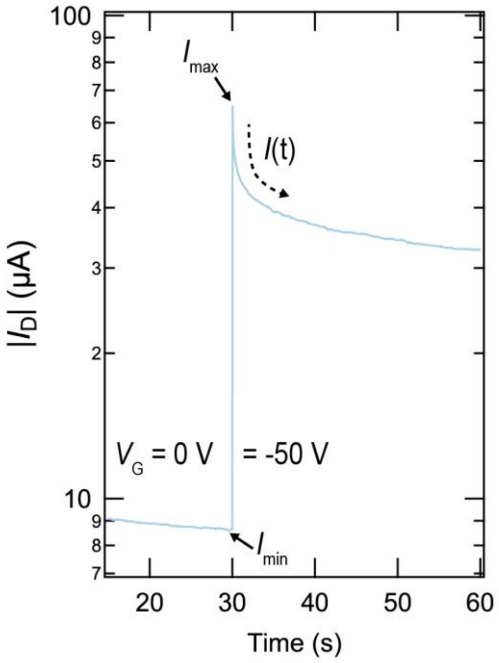

Figure S20. Method used to roughly correct the FET mobility for the effect of bias-stress current transients. The raw linear FET mobility ($\mu_{\text{lin,raw}}$) was calculated with the normal gradual channel approximation equation:

$$\left.\frac{dI_{\text{D}}}{dV_{\text{G}}}\right|_{V_{\text{SD}}} = \frac{WC_{\text{ox}}V_{\text{SD}}}{L}\mu_{\text{lin,raw}}$$

where the transconductance ($dI_{\text{D}}/dV_{\text{G}}$) was determined from the slope of transfer curves at V_{G} = -45 V for holes and +45 V for electrons. While acquiring the transfer curves, current transients cause a decay in the measured drain current and therefore an underestimation of the mobility. We approximated the transient-induced reduction in apparent hole mobility by calculating the drain current decay that occurs during the time required to measure the mobility (at a transfer curve sweep rate of 200 V/s, the required time is 225 ms for the reverse sweep mobility and 275 ms for forward sweep mobility). A transient correction factor F was calculated for each device at each temperature by measuring the transient produced by stepping V_{G} from 0 V to -50 V at V_{SD} = -15 V (see figure) and employing the following equation:

$$F = \frac{I(t) - I_{\text{min}}}{I_{\text{max}} - I_{\text{min}}}$$

where t = 225 or 275 ms, I_{min} is the minimum current at V_{G} = 0 V, and I_{max} is the maximum current at V_{G} = -50 V. The corrected mobility μ_{corr} was then calculated as $\mu_{\text{corr}} = \mu_{\text{lin,raw}}/F$. The data shown here were acquired from Device 1 at T = 290 K.

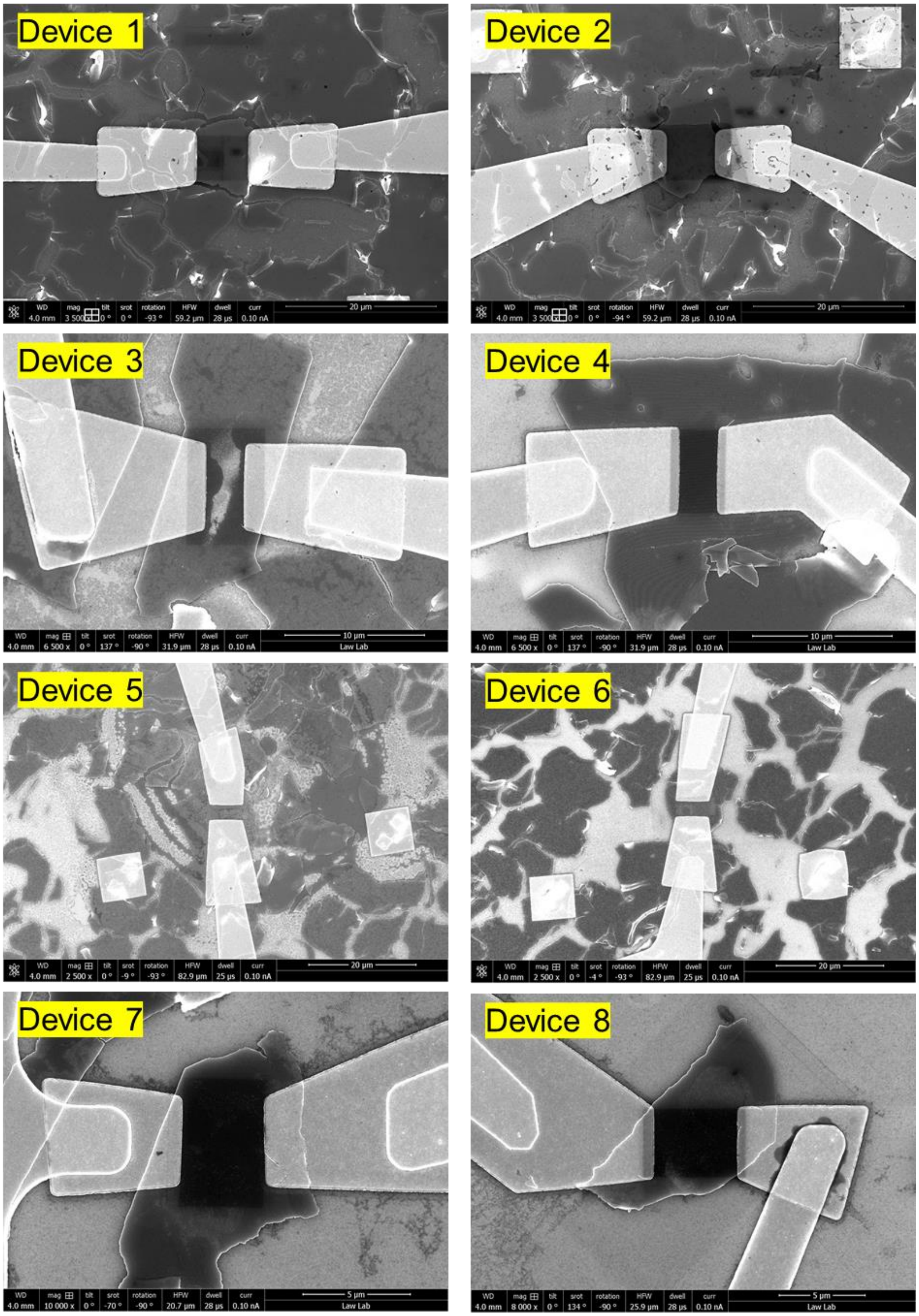

Figure S21. Plan-view SEM images showing the geometry of the epi-SL flake relative to the FET channel for all eight devices.

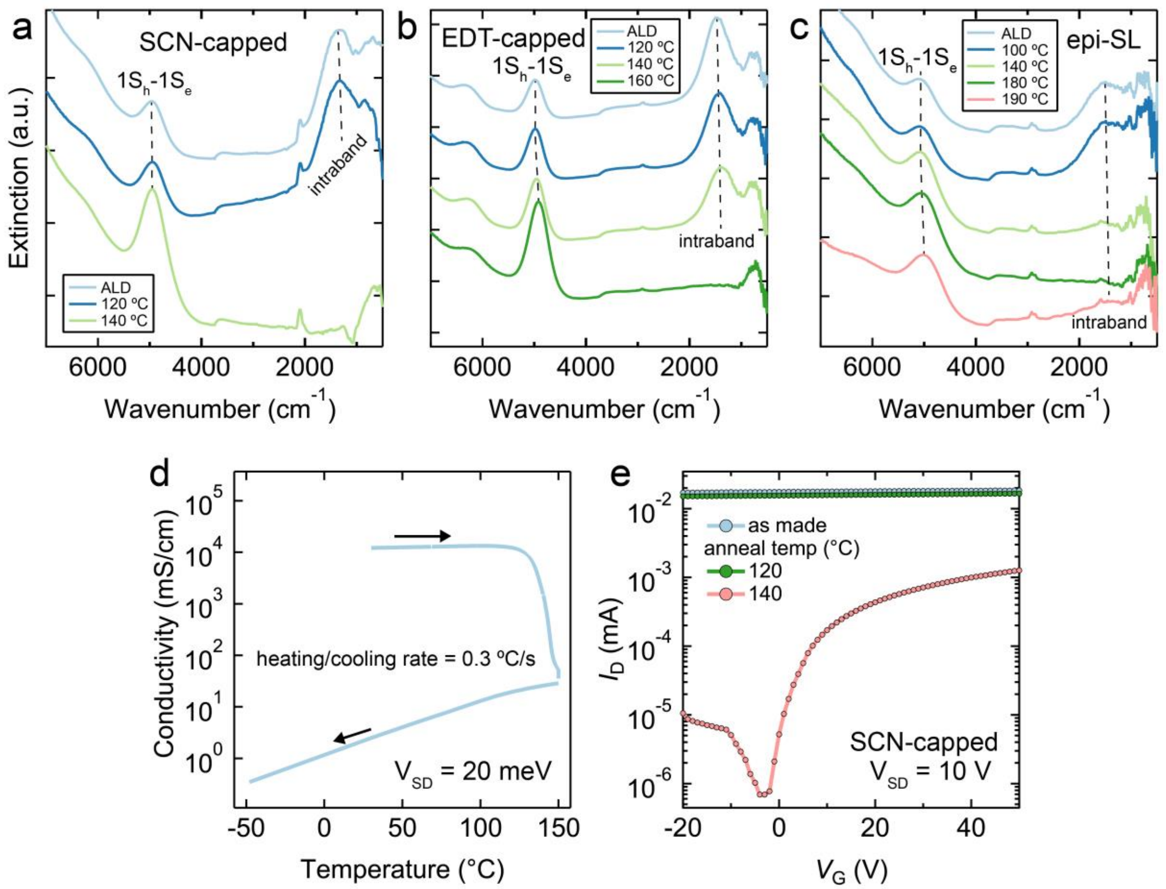

Figure S22. Effect of annealing on the doping of ALD-infilled PbSe QD films. All films were coated with 22 nm of ALD alumina deposited at 60 ºC. FTIR spectra of (a) an amorphous SCN-capped QD film (no long-range order), (b) an amorphous EDT-capped QD film, and (c) an EDA-treated epi-SL film similar to the samples studied in the main text. Each as-made film ("ALD") shows a significant $1S_e$-$1P_e$ intraband transition at ~1600 cm^{-1} and partial bleaching of the first exciton transition ("$1S_h$-$1S_e$") at ~5000 cm^{-1}, consistent with heavy *n*-doping of these films.[2] After annealing for three minutes at 130-190 °C in the glovebox, the intensity of the intraband transition was reduced or eliminated and the strength of the first exciton transition was increased, indicating a significant reduction in electron concentration due to donor passivation or compensation. The FTIR measurements in each of the panels (a-c) were performed at room temperature using a single sample. (d) In situ conductivity of an amorphous SCN-capped QD film FET (with dimensions L = 1000 μm, W = 25 μm, and thickness ≈ 50 nm) during heating from room temperature to 150 °C and then cooling to -50 °C at a ramp rate of 0.3 °C/second in a vacuum cryostat. The conductivity drops precipitously at ~140 °C, the same temperature at which the intraband transition disappears in the optical spectrum in (a). (e) Room-temperature transfer curves for a sibling sample of the FET in (d) showing a large decrease in I_D and recovery of strong gate modulation after annealing at 140 °C in the glovebox, consistent with a sharp reduction in the free electron concentration. This device is predominantly *n*-channel due to the SCN^- capping.

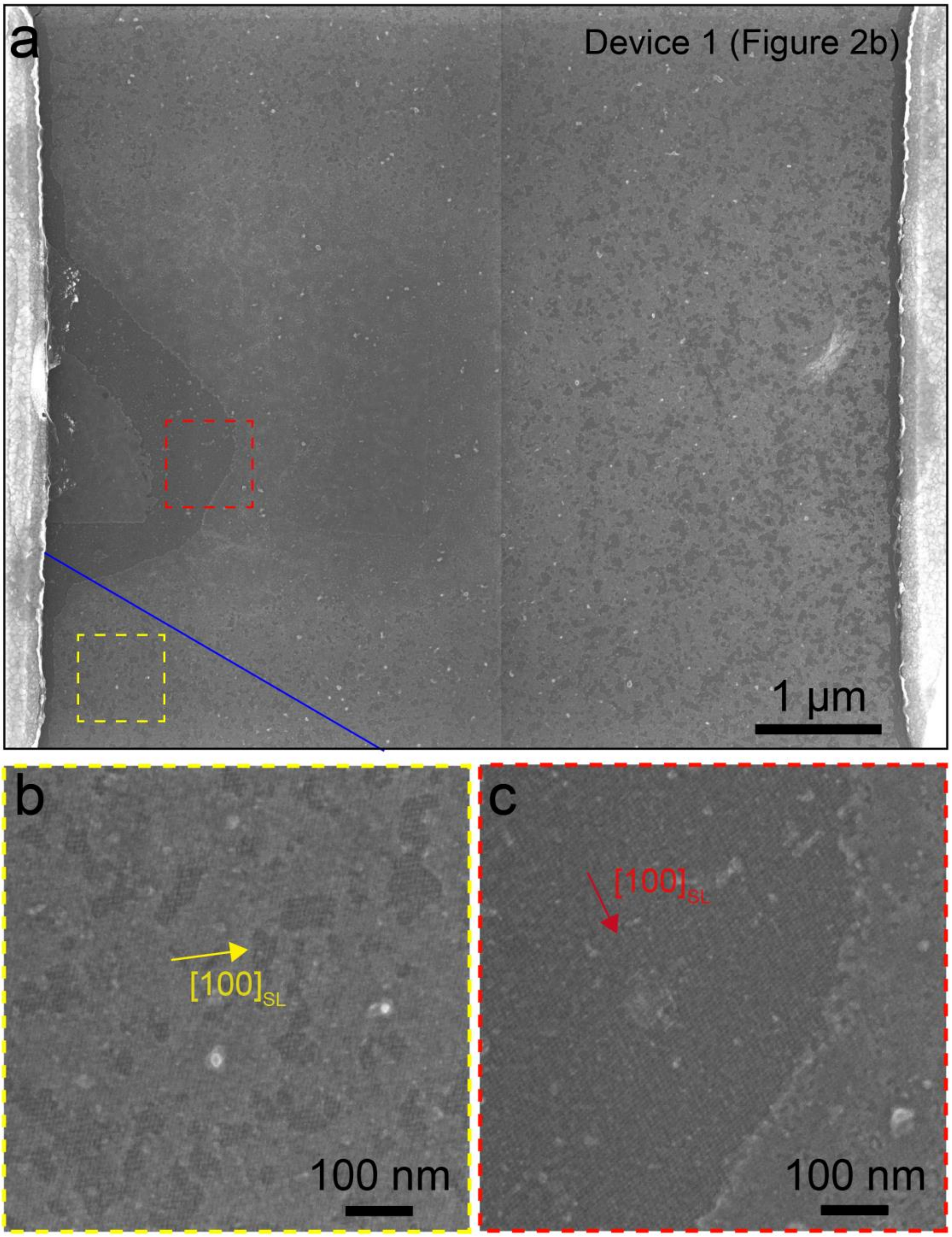

Figure S23. SEM images showing that the annealing treatments used for FET fabrication cause negligible QD sintering/ripening or epi-SL degradation. (a) Image reproduced from Figure 2b in the main text showing the entire channel of Device 1 after a post-mortem alumina etch. (b) Magnified view of part of the minor grain (dashed yellow box). The individual QDs are visible in the image and the $[100]_{SL}$ direction is indicated. (c) Magnified view of the major grain (dashed red box). The individual QDs are visible in the image and the $[100]_{SL}$ direction is indicated. Residual alumina is present in both images. Scale bars are 1 μm for (a) and 100 nm for (b) and (c).

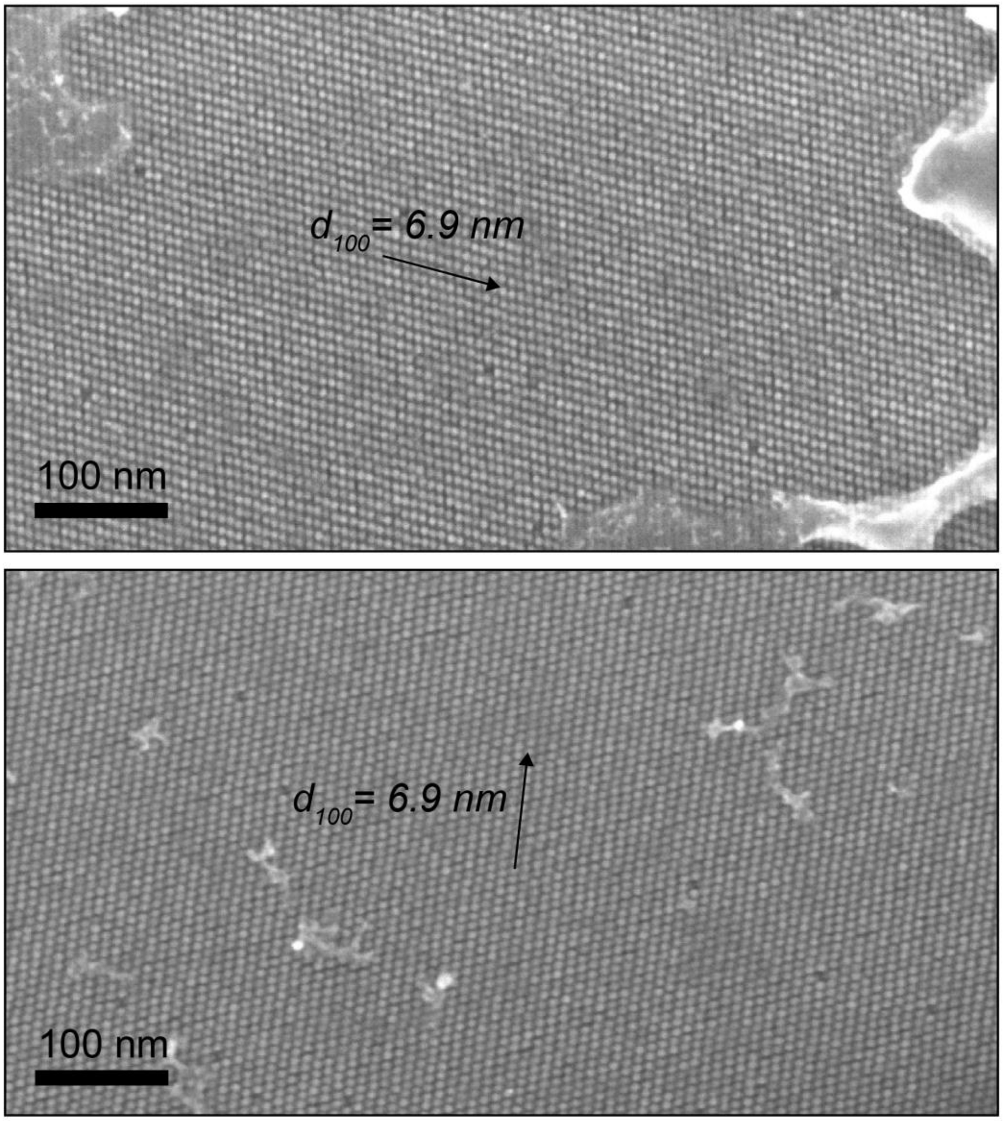

Figure S24. Additional high-resolution SEM images showing that the FET processing causes negligible QD sintering/ripening or epi-SL degradation. These images were acquired from a region just outside of the channel of Device 5 and highlight the pristine microstructure of epi-SLs subjected to the complete process of FET fabrication and post-mortem alumina removal. These images are clearer than those in Fig. S23 because the alumina removal is more complete outside of the device channels. SEM-induced carbon deposition tends to cause inhomogeneous alumina etching inside the device channels. Note that d_{100} refers to the center-to-center spacing of neighboring QDs along the close-packed $[100]_{SL}$ direction, as is reported in Table S1.

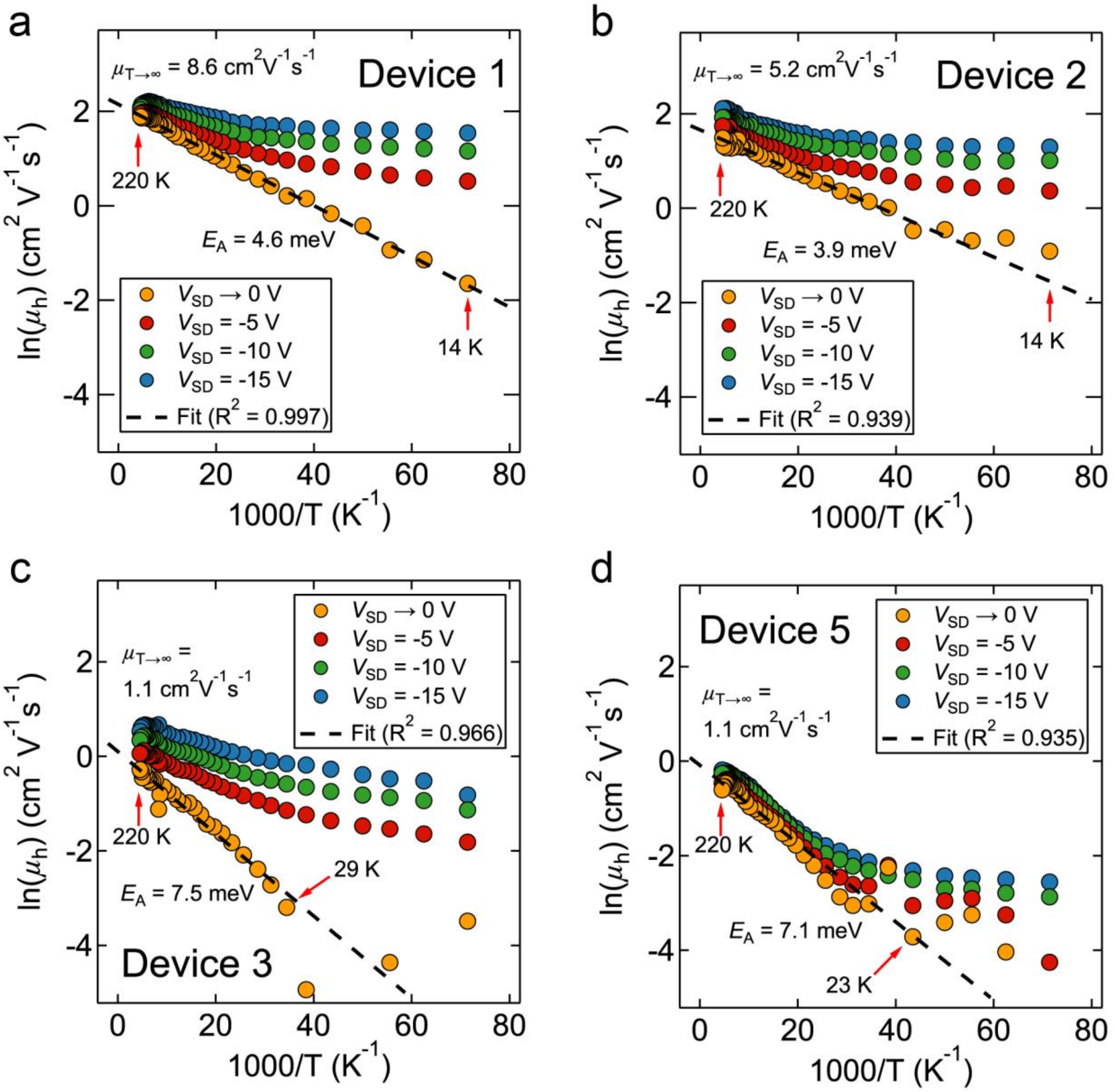

Figure S25. $\mu_{rev,corr}(T)$ for Devices 1, 2, 3 and 5 at different values of V_{SD}. In each panel, the reverse sweep, transient-corrected mobility is presented, as well as the field-free mobility extracted from linear fits of $\mu(V_{SD})$ at each temperature (see Figure S26). The field-free mobility trace is fit (black dashed line) between the temperature points noted on each panel. The appropriate range (indicated by red arrows on each plot) was selected based on visual inspection of the field-free mobility and $\mu(V_{SD})$ data. The slope and y-intercept of the fits were used to determine the activation energy (E_A) and mobility pre-factor (μ_∞) according to the function $\mu(T) = \mu_\infty \exp(-E_A/kT)$. The results of these fits are summarized in Table 1 of the main text.

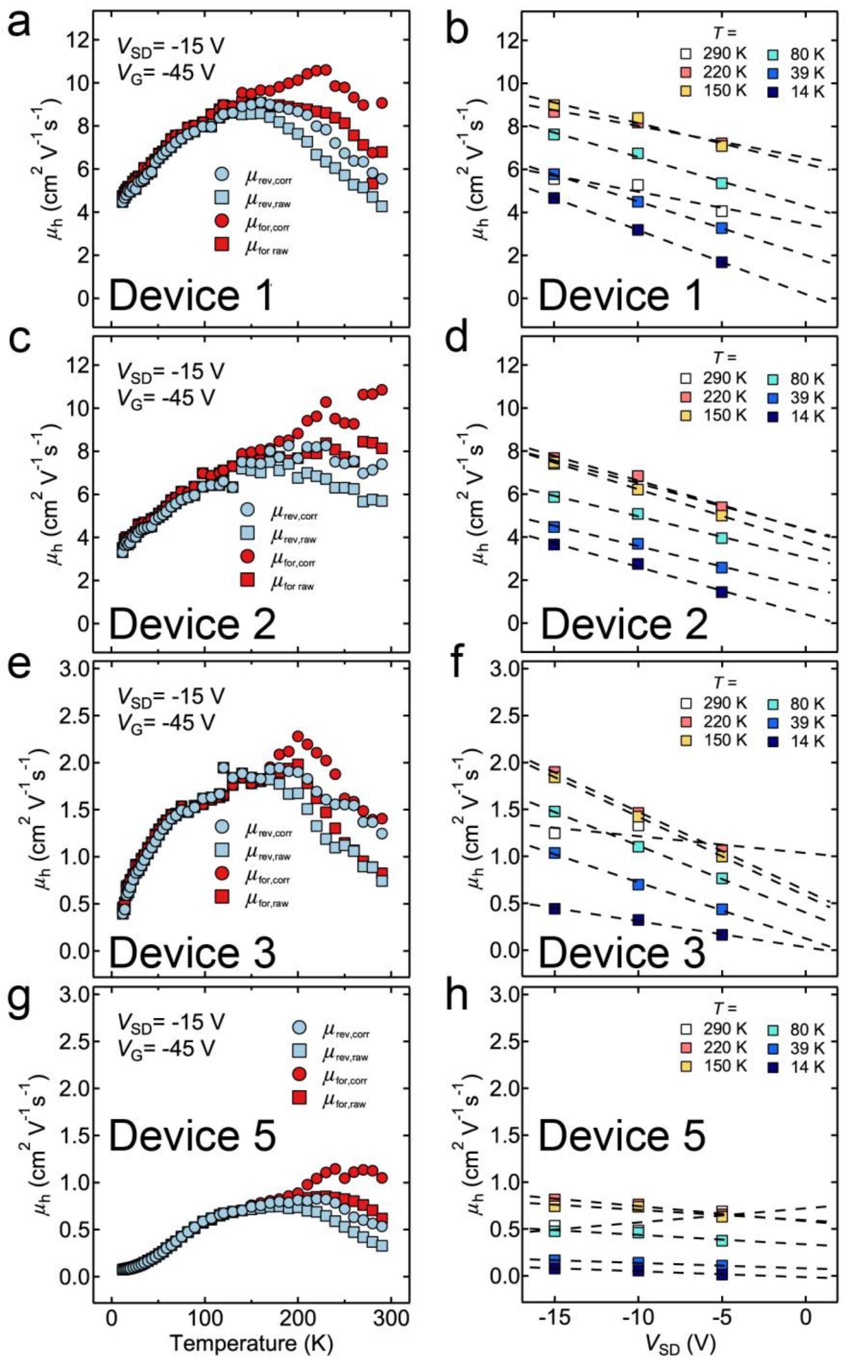

Figure S26. Impact of transient correction and V_{SD} on $\mu(T)$ for Devices 1, 2, 3 and 5. (a,c,e,g) Hole mobility (V_{SD} = -15 V, V_G = -45 V) of Devices 1, 2, 3, and 5 extracted from forward and reverse gate bias sweeps (200 V/s) without transient correction (squares) and with transient correction (circles). Panel (a) is reproduced from Figure 4 in the main text. (b,d,f,h) Linear fits of $\mu_{rev,corr}$ as a function of V_{SD} for Devices 1, 2, 3, and 5. Panel (b) is reproduced from Figure 4 in the main text, except here we include T = 220 and 290 K.

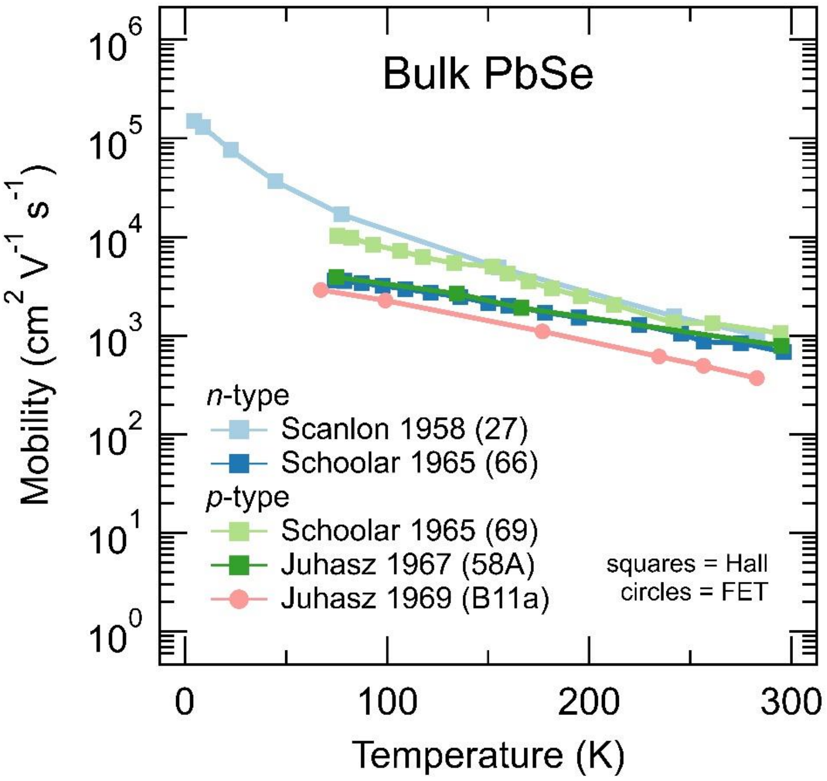

Figure S27. Compilation of literature values for the temperature-dependent mobility of bulk PbSe. In both Hall effect and field-effect transistor measurements, bulk PbSe exhibits anti-activated mobility ($d\mu/dT < 0$). All samples were moderately doped. Data were compiled from multiple sources,[3,4,5,6] as referenced in the legend, and include both *n*- and *p*-type crystals. In the legend, sample identifiers are provided along with the author surname and publication year.

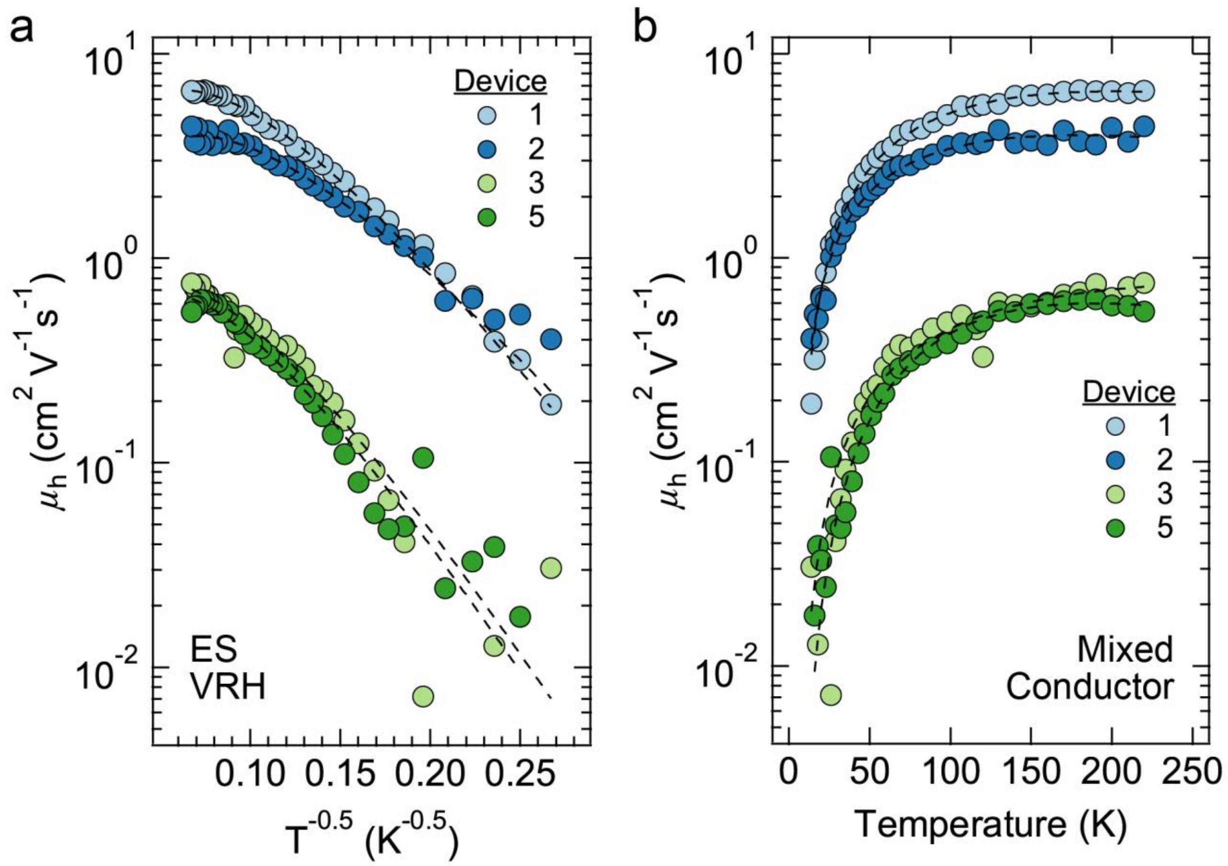

Figure S28. Additional $\mu_h(T)$ modeling of Devices 1, 2, 3 and 5. (a) ES-VRH fits with 1/*T* pre-factor (see main text). (b) Mixed conductor model fits following the work of Lan *et al*.[7]:

$$\mu(T)^{-1} = AT^{3/2} + B\exp\left[\left(\frac{T_{\mathrm{ES}}}{T}\right)^{\frac{1}{2}}\right]$$

Fits were performed on field-free mobilities between 14 and 220 K. The results of these fits are summarized in Table 1 of the main text.

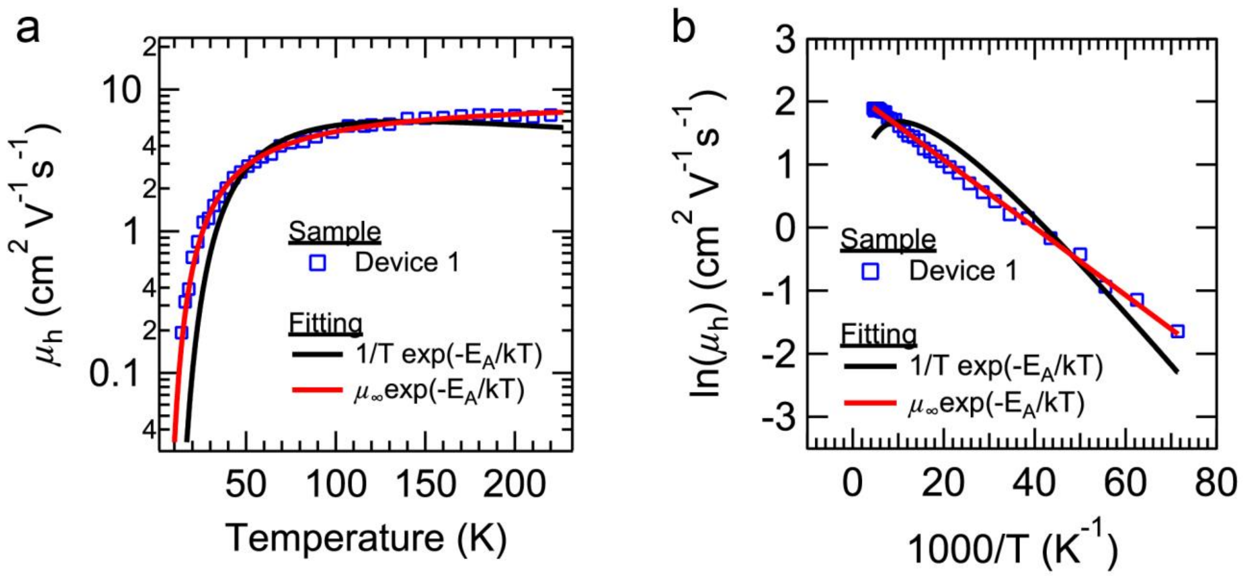

Figure S29. Comparison of nearest-neighbor hopping (NNH) fits with and without a temperature-dependent prefactor. (a) Log-linear plot and fits. (b) Natural logarithm of the mobility versus 1000/*T*. The fits without the 1/*T* prefactor are markedly better.

Supplementary Discussion 1: Device Fabrication.

Preparation of photopatterned substrates.
1. All epi-SL μ-FETs were fabricated on p^{++} (100)-oriented Si wafers coated with a 200 nm thick dry thermal oxide layer (Addison Engineering). The 4-inch wafers were first cleaned using 15 minute rounds of sonication in acetone, water, and isopropanol, followed by drying with a stream of compressed dry air (CDA) and 15 minutes of heating at 110 ºC. The wafers were then plasma cleaned in O_2/Ar for 10 minutes.

2. The wafers were photopatterned using the photomask shown in Figure S30a-c. Each wafer yields 52 copies of Die A (Fig. S30b). Die A contains a square ring array of twenty 800 µm square contact pads with tapered fingers that extend to a central 550 µm square region of interest dubbed the “playground” (Fig. S30c). The playground contains an 11 × 11 square array of 3 µm registration marks with a 50 µm pitch. Photopatterning began with the deposition of a photoresist primer layer by spin coating a 20% volume solution of hexamethyldisilazane (HMDS) in *p*-xylene at 3500 rpm for 120 seconds. A layer of Shipley S1808 photoresist was then spin cast onto the HMDS-treated wafer at 3500 rpm for 120 seconds. The photoresist layer was soft-baked at 90 ºC for 30 minutes. The photomask was then aligned over the center of the wafer and illuminated with an Ushio USH-508SA UV lamp for 2.6 seconds. The wafer was soaked in photoresist developer (Shipley Microposit MF-319) for 60 seconds, followed by soaking and gentle rinsing with water. The wafer was then dried for 5 minutes at 110 ºC and O_2/Ar plasma cleaned for 5 minutes.

3. 5 nm of chromium and 45 nm of gold were sequentially deposited in an MBraun thermal evaporation system inside of an N_2-filled glovebox (<0.5 ppm O_2). The base pressure of the evaporator was 5×10^{-6} mbar. The deposition rates for Cr and Au were 0.1-0.2 Å/s and 0.7 Å/s, respectively. A sample rotation stage (~10 rpm) was used during all metal depositions. Cr was deposited using Cr-plated W rods and Au was deposited from Au shot in a Mo boat.

4. Lift-off of the photoresist and metal was performed in air by sonicating in acetone. At this point, the wafers were diced by hand into 1.2 × 1.2 cm chips. Individual chips (rather than whole wafers) were used in subsequent processing steps. After dicing, the chips were cleaned as per Step 1. Figure S31 shows the device chips with photopatterned electrodes.

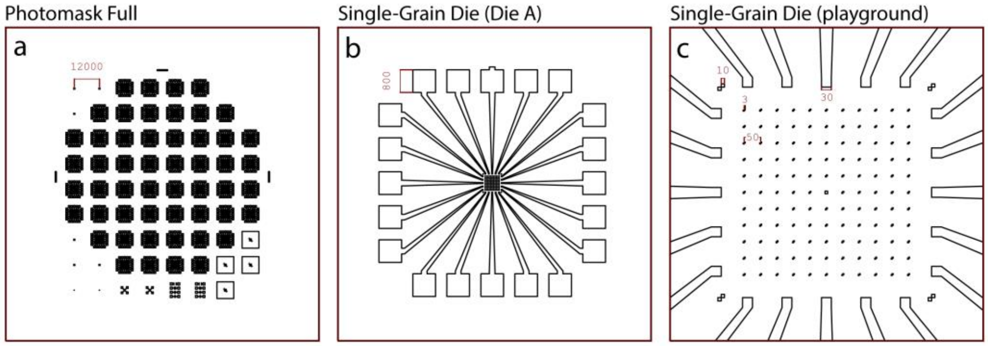

Figure S30. The μ-FET photomask and Die A. (a) Schematic the 5-inch photomask. (b) Die A. The diced chip size is 1.2 × 1.2 cm. (c) The “playground” at the center of Die A. All dimensions are in µm.

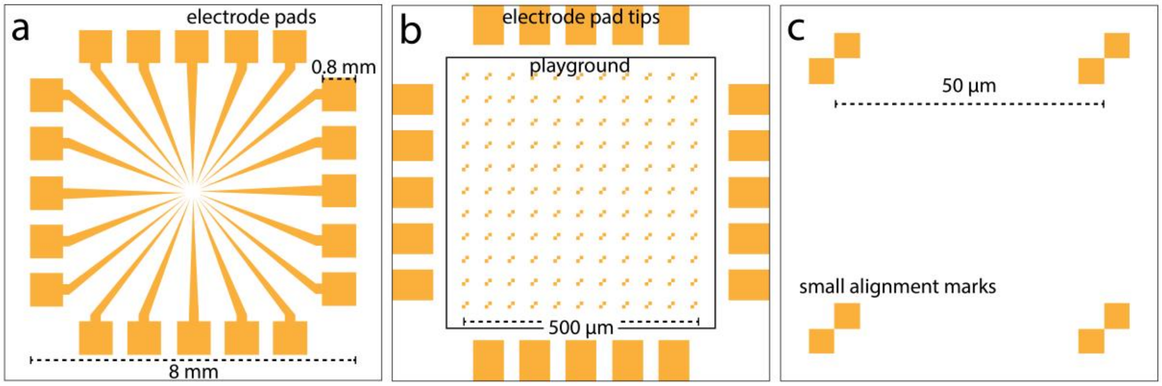

Figure S31. **The device chips with photopatterned electrodes.** (a) Overview. The electrodes (yellow) are 45 nm of Au on 5 nm of Cr. Each chip is ~1.2 × 1.2 cm. (b) View of the playground, with just the tips of the electrode pads shown. (c) Magnified view of a single quadrant of registration marks within the playground. These marks were used for fine alignment during electron beam lithography.

5. Each chip was photopatterned to expose only the 550 × 550 μm playground (Figure S32). Care was taken to align the photomask such that the tips of the electrode fingers were not exposed. Photopatterning and plasma descum were performed as per Step 2.

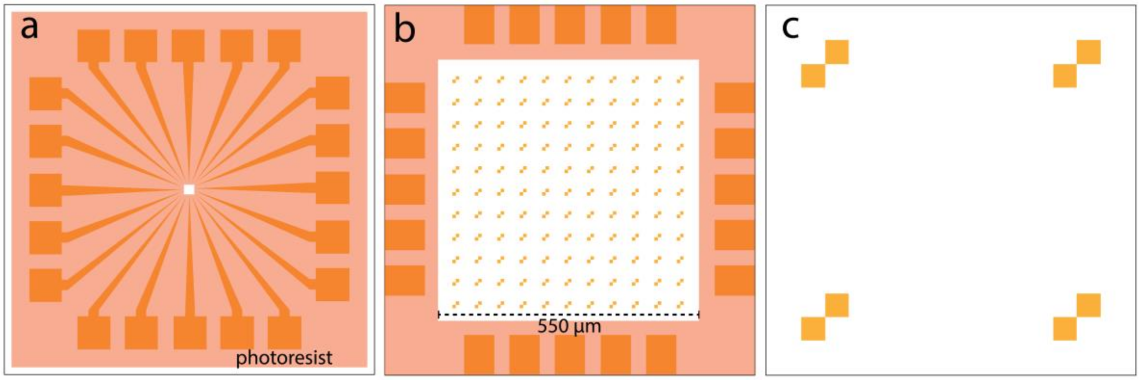

Figure S32. The chips after Step 5. The entire chip except the playground is coated with photoresist.

Epi-SL deposition, alumina deposition, imaging and alumina etching.

6. Prior to deposition of epi-SL film, the chips were functionalized to improve epi-SL adhesion by soaking them in 100 mM 3-MPTMS in toluene for 1 hour. This treatment was performed in the glovebox. The chips were then rinsed with neat toluene and blown dry with N_2.

7. Epi-SL fabrication was performed in N_2-filled gloveboxes with <0.5 ppm O_2. 60 μL of a 10 mg/mL (for Devices 1 and 2, the two thinner, higher-mobility devices) or 19 mg/mL (for Devices 3-8, the thicker, lower-mobility devices) solution of PbSe QDs dispersed in hexanes was pipetted onto 7 mL of ethylene glycol in a Teflon well. After depositing the QD solution, the well was immediately covered by a glass plate. The hexane was allowed to slowly evaporate

over 25-32 minutes, resulting in a smooth, dry oleate-capped QD film floating on the EG surface. 0.1 mL of 7.5 M ethylenediamine in acetonitrile was injected under an edge of the film and allowed to react for 20 seconds (Devices 1 and 2) or 30 seconds (Devices 3-8). The resulting epi-SL film was stamped onto the MPTMS-treated chip. The chips were then doused with acetone, causing the photoresist film to dissolve. The chips were thoroughly cleaned with acetone, followed by rinsing with acetonitrile. This process resulted in epi-SL film deposited only in the playground.

9. Each chip was then transferred air-free to an in-glovebox atomic layer deposition (ALD) system and coated with 11 nm of amorphous alumina using trimethylaluminum and water at a substrate temperature of 60 ºC. Precursors were introduced to the ALD chamber using computer-controlled diaphragm valves in-line with a 130 sccm stream of N_2 carrier gas. Pulse and purge times were 20 ms and 55 seconds, respectively, for both precursors. This step resulted in ALD coating of the entire chip, including infilling and overcoating of the epi-SL film in the playground. Note that the epi-SL film was not exposed to air because the ALD system is in the glovebox.

10. Each chip was then imaged by SEM to locate and map the best epi-SL grains for electron beam lithography. Care was taken to minimize exposure to (*i*) air while transferring chips between the glovebox and SEM and (*ii*) the electron beam during imaging. Control experiments showed that μ-FETs capped with 11 nm of alumina showed minimal changes in electrical characteristics (<5% change in mobility) following several days of storage in air.

11. After SEM mapping, an additional 22 nm of alumina was deposited on each chip (33 nm in total) to guarantee the long-term stability of the epi-SL film. If needed, the chip was rinsed in water and IPA and blown dry. Figure S33 shows the chip after this step.

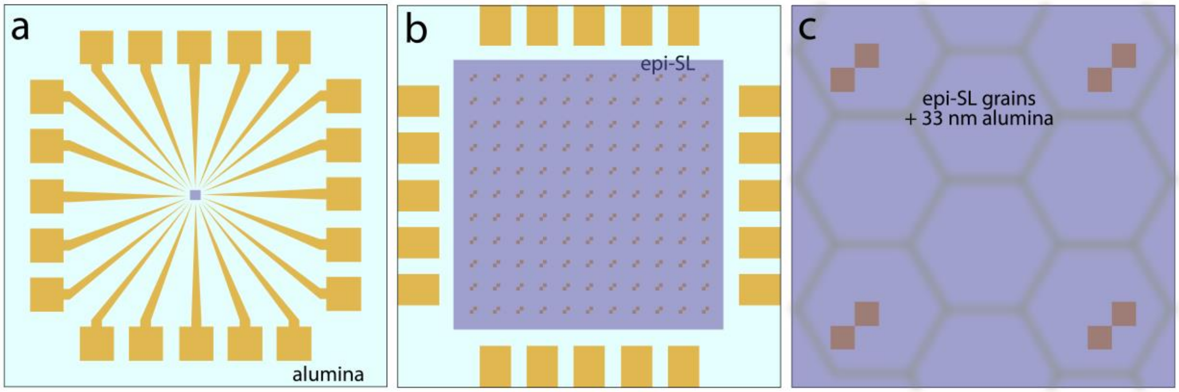

Figure S33. The chips after Step 11. (a) Overview. ALD alumina (light blue) covers the entire chip. The ALD-infilled epi-SL (dark blue) is in the playground. (b) View of the playground. The ALD-infilled epi-SL film is isolated from the electrodes. (c) Magnified view of a single quadrant of registration marks within the playground. The hexagonal tiling represents epi-SL grains.

12. Each chip was photopatterned again to protect a 600 × 600 μm square centered on the playground and the area outside of the electrodes with photoresist. Photopatterning and plasma descum were performed as per Step 2. At this point, the epi-SL film is protected by a sacrificial layer of photoresist, while the electrodes are free of photoresist.

13. Alumina on the exposed areas of the chip was etched for 55 minutes in an aqueous etching solution consisting of 150 mg glycerine (50 mM) and 51.2 mg of NaOH (32 mM) in 40 mL of H_2O (pH ~10). Freshly-made solutions were required to consistently achieve the desired etch rate (~0.6 nm/min), so fresh solution was prepared for each chip. After alumina etching, the chips were rinsed with water, acetone, water, and IPA, then blown dry. The acetone rinse removes the protective photoresist on the epi-SL film. Figure S34 shows the chip after this step.

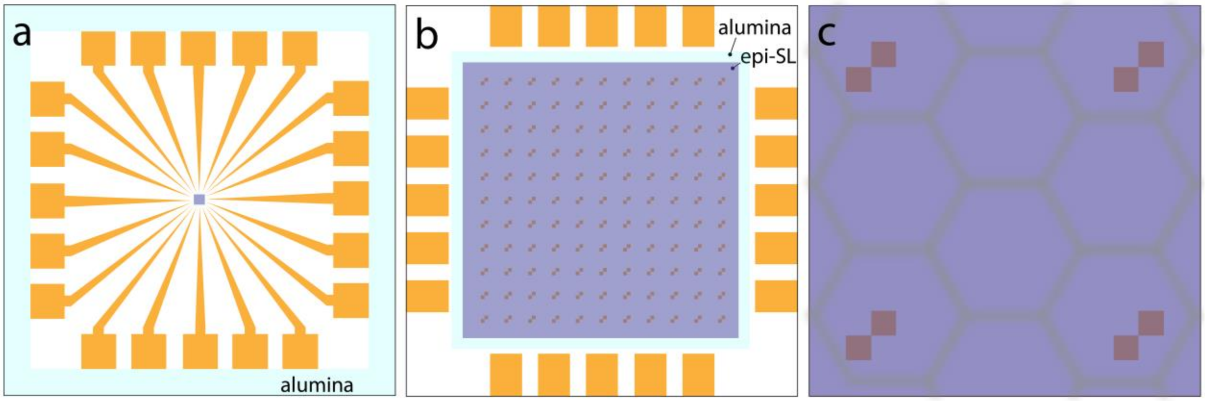

Figure S34. The chips after Step 13. (a) Overview. Alumina is etched from the electrodes but remains on the epi-SL film and the edge of the chip. (b) View of the playground. Alumina covers only the epi-SL film and has been removed from the electrode tips. (c) Magnified view of a single quadrant of registration marks within the playground.

Electron beam lithography to write coarse electrodes.

We used two rounds of electron beam lithography (EBL) and metallization to write electrodes from the finger electrodes to individual epi-SL grains. EBL was first used to write coarse electrodes extending from the finger electrodes to within several μm of the epi-SL grain of interest. A second round of EBL was used to write fine electrodes from the coarse electrodes to the epi-SL grain. Two rounds of EBL were used so that electrical vias could be etched through the alumina to make selective contact to the epi-SL grain of interest.

14. Each chip was coated with electron beam resist (PMMA) by spin coating at 2750 rpm for 60 seconds, then baked on a hotplate at 150 ºC for 3 minutes in air. Coarse electrodes were designed using DesignCAD Express 16 and written using Nanometer Pattern Generation System (NPGS) software. The e-beam voltage and current were 30 kV and ~1.6 nA, respectively. The latter was verified prior to each run using a Faraday cup and picoammeter. The absolute charge dose was specified at 550 $\mu C/cm^2$. After e-beam exposure, the chip was developed for 40 seconds in a 1:3 mixture of MIBK and IPA, rinsed with neat IPA and blown dry.

15. 5 nm of chromium and 45 nm of gold were sequentially deposited as per Step 3.

16. Following metal deposition, lift-off was performed by soaking the substrate in acetone for 3-5 hours, followed by vigorous rinsing with acetone. The chip was gently rinsed in acetone, water, and IPA, then blown dry. Figure S35 shows the chip after this step.

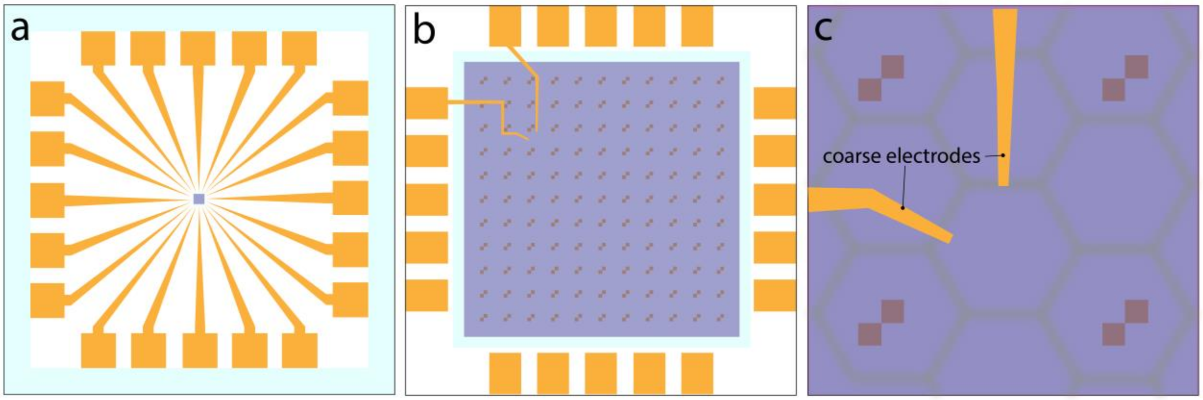

Figure S35. The chips after Step 16. (a) Overview. (b) View of the playground. Coarse electrodes have been written from the finger electrodes to within a few μm of the epi-SL grain of interest. Note that only one set of coarse electrodes is shown. (c) Magnified view of a single quadrant of registration marks within the playground.

Electron beam lithography to write fine electrodes.

17. Each chip was again coated with PMMA as per Step 14. Fine electrodes from the course electrodes to the epi-SL grain were designed and patterned using DesignCAD Express 16 and the NPGS software. The e-beam voltage and current were 30 kV and ~50 pA, respectively. The absolute charge dose was specified at 550 μC/cm^2. After exposure, the chip was developed for 40 seconds in a 1:3 mixture of MIBK and IPA, rinsed with neat IPA and blown dry.

18. The chip was transferred into the glovebox and through-alumina vias were etched (55 minutes of etching). For this step, de-oxygenated water (prepared by three freeze-pump-thaw cycles) was used. The etch solution consisted of 37.5 mg glycerine (50 mM) and 12.8 mg of NaOH (32 mM) in 10 mL of H_2O (pH ~10.2). After etching, the chips were rinsed vigorously with neat H_2O, then blown dry. Figure S36 shows the chip after this step.

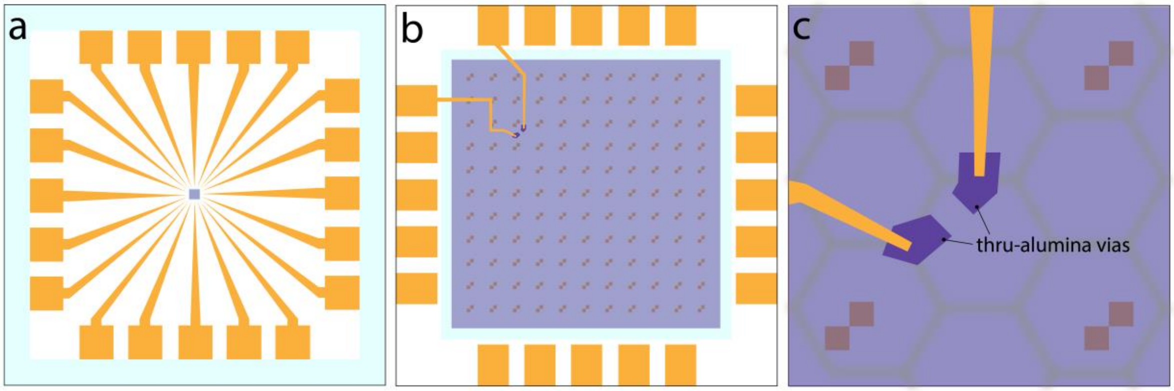

Figure S36. The chips after Step 18. (a) Overview. (b) View of the playground. Through-alumina vias (purple) formed at the tips of the coarse electrodes define the shape of the fine electrodes. The epi-SL grain is exposed at the bottom of the vias. (c) Magnified view of a single quadrant of registration marks within the playground. Through-alumina vias span connect the coarse electrode tips to the edges of the epi-SL grain of interest.

19. 5 nm of chromium and 45 nm of gold were sequentially deposited as per Step 3.

20. Lift-off was carried out by soaking the substrate in acetone for 3-6 hours followed by agitation of the solution with a glass pipette. This process was performed in an N_2-filled glovebox. The chip was rinsed vigorously in acetone and acetonitrile, then blown dry. A completed chip is presented in Figure S37. Although only one device is shown, there were typically 5-10 working devices on each chip. Due to time constraints, most of these devices have not yet been measured in detail.

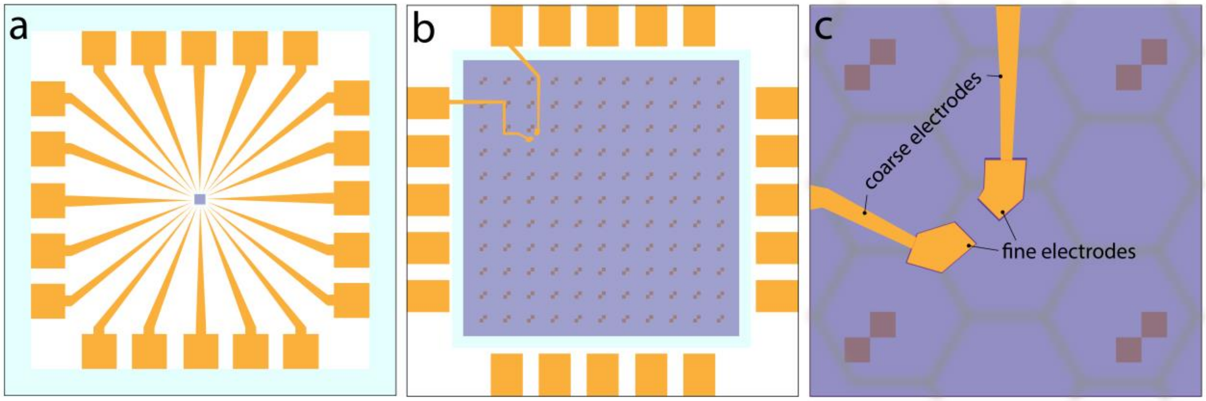

Figure S37. The completed epi-SL μ-FETs. (a) Overview. (b) View of the playground. Fine electrodes have been deposited into the vias to achieve intimate electrical contact with the epi-SL grain. The device area is defined by the region between the fine electrodes. (c) Magnified view of a single quadrant of registration marks within the playground. Fine electrodes have been formed at the tips of the coarse electrodes.

Additional encapsulation and electrical measurements.

21. 11 or 22 nm of additional alumina was deposited by ALD to further stabilize the devices. We did not observe a systematic difference in FET electrical behavior between these two overlayer thicknesses.

22. The devices on each chip were measured first in the glovebox at room temperature and then in a cryostat located outside of the glovebox.

Postmortem SEM imaging of the FET channels.

23. Following electrical measurements, each chip was transferred to the glovebox and the alumina encapsulation was etched away as per Step 18. The individual epi-SL μ-FETs were then imaged to determine the microstructure of the epi-SL grain(s) in the FET channel.

Supplementary Discussion 2: Estimating Site Disorder and Charging Energy.

Determination of the energy disorder.

Fitting the optical extinction spectrum of our PbSe QDs dispersed in TCE using the empirical relationship of Moreels *et. al.*[1] yields a first-exciton ($1S_h$-$1S_e$) absorption energy of 0.654 eV and a standard deviation of the Gaussian absorption peak of 20 meV. From this, we conclude that the standard deviation of the $1S_e$ (or 1 S_h) states of the dispersed QDs is 10 meV. This value describes QDs that are physically isolated (in solution). However, the $1S_e$ (or 1 S_h) variance is expected to increase upon formation of the epi-SL, so 10 meV is a lower bound to the site energy disorder ($\Delta\alpha$) of QDs in the epi-SL.

Calculation of the static dielectric constant of ALD-infilled epi-SLs.

Using the Penn approximation[8] with a bulk PbSe bandgap of 0.26-0.29 eV and bulk dielectric constant of 200-250,[9,10] we estimated the static dielectric constant of our 6.9 nm PbSe QDs isolated in vacuum (ε_{QD}) to be 80 ± 20. The Bruggeman equation[11] was then used to estimate an effective static dielectric constant for the ALD-infilled epi-SLs (ε_{eff}) of 36 ± 12. This value assumes a QD volume fraction in the alumina-infilled epi-SLs of 0.55 (as determined from the known superlattice unit cell), 75% of the void space filled with alumina (the rest N_2), and a dielectric constant for the alumina of 1-10.[12,13]

Calculation of QD charging energy in the ALD-infilled epi-SLs.

This analysis follows that of Liu *et al*.[14,15] The charging energy is given as:

$$E_C = e^2/[2(C_S + 6C_M)]$$

where C_S is the self-capacitance of a QD of radius r in an epi-SL of effective dielectric constant ε_{eff}, given as:

$$\frac{1}{C_S} = \frac{1}{4\pi\varepsilon_0 r}\left(\frac{\varepsilon_{QD} - \varepsilon_{eff}}{\varepsilon_{QD}\varepsilon_{eff}}\right) + \frac{0.94}{4\pi\varepsilon_{QD}\varepsilon_0 r}\left(\frac{\varepsilon_{QD} - \varepsilon_{eff}}{\varepsilon_{QD} + \varepsilon_{eff}}\right)$$

and the C_M is the mutual capacitance between adjacent pairs of QDs:

$$C_M \cong 2\pi\varepsilon_0\left(\frac{\varepsilon_{QD}\varepsilon_{eff}}{\varepsilon_{QD} - \varepsilon_{eff}}\right) r \ln\left[\frac{2r + d}{d}\right]$$

where ε_0 is the permittivity of free space, r is the QD radius (3.45 nm), d is the length of the inter-QD epitaxial neck (assumed to be 0.6 nm, or 2 atomic layers along $<100>_{PbSe}$), and the factor of six in the expression for E_C accounts for the six nearest neighbors in the 3D epi-SL structure. Following this analysis, we estimate a charging energy of only ~0.3 meV. Using even the most conservative set of reasonable assumptions (e.g., ε_{QD} = 250, ε_{eff} = 5, and five nearest neighbors in the 2D FET channel), we find that E_C is less than 4.5 meV.

Supplementary Discussion 3: Comparison of Charge Transport Models.

Consideration of Variable-Range Hopping and Quantum Percolation Scaling.

To fit single-grain FET mobilities, individual Arrhenius NNH and ES-VRH fits were evaluated, as well as quantum percolation scaling (QPS) fits which include both an insulating and conducting channel.[16] For the QPS fits, the mean field exponent $m = 1$ was chosen following our previous work.[16] To place this fitting work into physical context, we calculated the localization length in the Efros-Shklovskii picture, and found it to be 2-4 nm, as reported in the main text. This indicates that holes are largely localized to a single QD. Moreover, in the ES picture, the average length d of the "variable range" hops can be calculated: $d \sim d_0(E_C/k_BT)^{1/2}$, where d_0 is the diameter of a QD, k_B is Boltzmann's constant, and the charging energy $E_C \sim 0.3$ meV, as calculated in the previous section. The experimental temperatures range between 12 and 220 K, and so d/d_0 spans 0.29 and 0.01. In the upper limit of charging energy ($E_C = 4.5$ meV) d/d_0 spans between 4.3 and 0.23, and is less than unity above $T = 53$ K. Taking $E_C = 0.3$ meV, this means that for most devices over most of the temperature range, the average hopping distance is less than a single QD diameter. This is entirely consistent with the localization length also being smaller than a single QD. Together, these considerations suggest that the dominant hopping process involve only nearest neighbors, and thus variable-range hopping models are not appropriate to analyze our data. However, if E_C is significantly underestimated, ES-VRH may occur at low temperatures.

A Brief Note on Metallic Conduction in 2-Dimensional Systems.

It is well established that in two-dimensional (2D) non-interacting electron systems, all electron states are localized at $T = 0$, a phenomenon called "Anderson localization". Therefore, the wisdom of describing our samples as even partially "metallic" could be questioned. To place our analysis in proper context, it is important to recall here that there are noted limitations to the dominance of Anderson localization.

First, 2D localization manifests itself only at very low temperatures. Most experimental work on Anderson localization must measure transport at $T \leq 1$ K to detect signs of localization, and even there the localization shows up in the form of small, fractional changes, not as a dramatic suppression of the conductivity. Therefore, since most of our data was taken at tens of K up to 220 K, localization effects are expected to be either undetectable, or on the percent scale.

Second, the localization is complete only for non-interacting electrons, whereas our systems are characterized by a finite Coulomb interaction strength.

Third, we are modeling transport across metallic clusters of finite size. In weakly disordered systems like ours, the localization length can be quite long, and thus the signs of localization on the finite size clusters may be negligible.

Given the above considerations, it is a reasonable terminology to conceptualize the transport in Coulomb-interacting 2D metallic clusters in the 10-220 K range as appearing metallic, even if acknowledging that at $T = 0$ on macroscopic scales the electron states may be localized, especially if the Coulomb interaction is absent.

Supplementary Discussion 4: Quantification of $\mu_h(T)$ Fits.

Goodness of fits for $\mu_h(T)$ of Devices 1, 2, 3, and 5 were evaluated by determining the residual sum of squares (RSS). The results for all fits are compiled below (Table S2). Lower values of RSS indicate better agreement between fits and measured data. The QPS-Arrhenius fit with s = 1/2 shows the lowest RSS values across all devices and so we present these fits in Figure 5b in the main text. Both the s = 0 QPS-Arrhenius and QPS-ES fits show lower or comparable RSS values compared to the pure insulating (non-QPS) models, indicating that the inclusion of a metallic channel provides a better fit to the data.

Table S2. RSS values for fits of $\mu_h(T)$ using several models.

	Non-QPS		QPS-Arrhenius		QPS-ES		QPS-Mod. ES	
Device	Arrhenius	Modified ES	$s=0$	$s=½$	$s=0$	$s=½$	$s=0$	$s=½$
1	0.649	0.448	0.535	0.287	0.535	0.287	7.472	2.758
2	1.398	1.272	1.280	1.228	1.280	1.228	4.275	2.290
3	0.074	0.081	0.074	0.072	0.074	0.083	0.133	0.095
5	0.036	0.030	0.022	0.021	0.022	0.021	0.036	0.027

The value of s indicates the conductivity exponent used in QPS fits. "Arrhenius" refers to a simple Arrhenius function with no $1/T$ pre-factor (see Figure 5a in the main text). "Modified ES" refers to the Efros-Shklovskii VRH model with a $1/T$ pre-factor. "QPS-Arrhenius" refers to the Quantum Percolation Scaling model with mixed metallic and Arrhenius hopping conduction (see Figure 5b in the main text). "QPS-ES" refers to the Quantum Percolation Scaling model with mixed metallic conduction and ES-VRH hopping conduction, which reduces for s = 0 to the mixed conduction model presented in Figure S28b. "QPS-Mod. ES" refers to the Quantum Percolation Scaling model with mixed metallic and ES-VRH hopping conduction, where the ES-VRH term includes a $1/T$ pre-factor.

References

[1] Moreels, I.; Lambert, K.; De Muynck, D.; Vanhaecke, F.; Poelman, D.; Martins, J. C.; Allan, G.; Hens, Z. Composition and Size-Dependent Extinction Coefficient of Colloidal PbSe Quantum Dots. *Chem. Mater.* **2007**, *19* (25), 6101–6106. https://doi.org/10.1021/cm071410q.

[2] Abelson, A.; Qian, C.; Salk, T.; Luan, Z.; Fu, K.; Zheng, J.-G.; Wardini, J. L.; Law, M. Collective Topo-Epitaxy in the Self-Assembly of a 3D Quantum Dot Superlattice. *Nat. Mater.* **2020**, *19* (1), 49–55. https://doi.org/10.1038/s41563-019-0485-2.

[3] Allgaier, R. S.; Scanlon, W. W. Mobility of Electrons and Holes in PbS, PbSe, and PbTe between Room Temperature and 4.2 K. *Phys. Rev.* **1958**, 111 (4), 1092–1037. https://doi.org/10.1103/PhysRev.111.1029.

[4] Zemel, J. N.; Jensen, J. D.; Schoolar, R. B. Electrical and Optical Properties of Epitaxial Films of PbS, PbSe, PbTe, and SnTe. *Phys. Rev.* **1965**, 140 (1A), 330–342. https://doi.org/10.1103/PhysRev.140.A330.

[5] Egerton, R. F.; Juhasz, C. Epitaxial Films of PbTe, PbSe, and PbS Grown on Mica Substrates. *Br. J. Appl. Phys.* **1967**, 18, 1009–1011. https://doi.org/10.1088/0508-3443/18/7/417.

[6] Egerton, R. F.; Juhasz, C. Field-Effect Measurements on Epitaxial PbTe and PbSe Films. *J. Phys. D: Appl. Phys.* **1969**, 2 (7), 975–984. https://doi.org/10.1088/0022-3727/2/7/304.

[7] Lan, X.; Chen, M.; Hudson, M. H.; Kamysbayev, V.; Wang, Y.; Guyot-Sionnest, P.; Talapin, D. V. Quantum Dot Solids Showing State-Resolved Band-like Transport. *Nat. Mater.* **2020**, *19* (3), 323–329. https://doi.org/10.1038/s41563-019-0582-2.

[8] Sharma, A. C. Size-Dependent Energy Band Gap and Dielectric Constant within the Generalized Penn Model Applied to a Semiconductor Nanocrystallite. *J. Appl. Phys.* **2006**, *100* (8), 084301. https://doi.org/10.1063/1.2357421.

[9] Allgaier, R. S.; Scanlon, W. W. Mobility of Electrons and Holes in PbS, PbSe, and PbTe between Room Temperature and 4.2 ˚K. *Phys. Rev.* **1958**, *111* (4), 1029–1037. https://doi.org/10.1103/PhysRev.111.1029.

[10] Kang, I.; Wise, F. W. Electronic Structure and Optical Properties of PbS and PbSe Quantum Dots. *I. Opt. Soc. Am. B.* **1997**, 14, 1632–1646. https://doi.org/10.1364/JOSAB.14.001632.

[11] Grinolds, D. D. W.; Brown, P. R.; Harris, D. K.; Bulovic, V.; Bawendi, M. G. Quantum-Dot Size and Thin-Film Dielectric Constant: Precision Measurement and Disparity with Simple Models. *Nano Lett.* **2015**, *15* (1), 21–26. https://doi.org/10.1021/nl5024244.

[12] Gieraltowska, S.; Wachnicki, Ł.; Witkowski, B. S.; Godlewski, M.; Guziewicz, E. Properties of Thin Films of High-k Oxides Grown by Atomic Layer Deposition at Low Temperature for Electronic Applications. *Optica Applicata* **2013**, 43(1), 17–25. https://doi.org/10.5277/oa130102.

[13] Acharya, J.; Wilt, J.; Liu, B.; Wu, J. Probing the Dielectric Properties of Ultrathin Al/Al2O3/Al Trilayers Fabricated Using in Situ Sputtering and Atomic Layer Deposition. *ACS Appl. Mater. Interfaces* **2018**, *10* (3), 3112–3120. https://doi.org/10.1021/acsami.7b16506.

[14] Liu, Y.; Gibbs, M.; Puthussery, J.; Gaik, S.; Ihly, R.; Hillhouse, H. W.; Law, M. Dependence of Carrier Mobility on Nanocrystal Size and Ligand Length in PbSe Nanocrystal Solids. *Nano Lett.* **2010**, *10* (5), 1960–1969. https://doi.org/10.1021/nl101284k.

[15] Lannoo, M.; Delerue, C.; Allan, G. Screening in Semiconductor Nanocrystallites and Its Consequences for Porous Silicon. *Phys. Rev. Lett.* **1995**, *74* (17), 3415–3418. https://doi.org/10.1103/PhysRevLett.74.3415.

[16] Qu, L.; Vörös, M.; Zimanyi, G. T. Metal-Insulator Transition in Nanoparticle Solids: Insights from Kinetic Monte Carlo Simulations. *Sci. Rep*. **2017**, *7* (1), 7071. https://doi.org/10.1038/s41598-017-06497-1.
